# Assessing the impact of COVID-19 on STEM (science, technology, engineering, mathematics) researchers in India

**DOI:** 10.12688/wellcomeopenres.17853.2

**Published:** 2023-07-11

**Authors:** Nikita Mehta, Vedika Inamdar, Arathy Puthillam, Shivani Chunekar, Hansika Kapoor, Anirudh Tagat, Deepa Subramanyam

**Affiliations:** 1 Department of Psychology, Monk Prayogshala, Mumbai, Maharashtra, 400072, India; 2 Department of Sociology, Monk Prayogshala, Mumbai, Maharashtra, 400072, India; 3 Department of Economics, Monk Prayogshala, Mumbai, Maharashtra, 400072, India; 4 National Centre for Cell Science, SP Pune University, Pune, Maharashtra, 411007, India

**Keywords:** COVID-19, STEM researchers, gender, health, productivity, disruption

## Abstract

**Background: **The coronavirus disease 2019 (COVID-19) pandemic and the nationally mandated lockdown has resulted in facility closures, decreased laboratory activities, and shifting to remote working. The effects of the pandemic have spread across all professions, including academia. Hence, the present study aims to understand the extent of the impact of the COVID-19 pandemic on STEM (science, technology, engineering, mathematics) researchers and stakeholders in India.

**Methods: **The study employed a mixed method design. Both quantitative (survey) and qualitative (interview) methods were used to gain a comprehensive understanding on the impact of the COVID-19 pandemic on STEM (science, technology, engineering, mathematics) early career researchers (ECRs), graduate students, Heads of Institutes, suppliers of scientific equipment, funders, and other stakeholders in India.

**Results: **A total of 618 researchers completed the survey, and 24 stakeholders were interviewed for this study. Our findings highlight the importance of institutional and social support for mental well-being and scientific productivity among researchers, especially during the pandemic. It also shows the impact of the disruptions in grant disbursals on research activities of scientists. Further, the gendered impact between these relationships was also noted, all of which hint at a need for structured reform within STEM.

**Conclusions: **The study highlights the various challenges faced by early career researchers, and STEM scientists at various positions in their careers during the COVID-19 restrictions in India.

## Introduction

The coronavirus disease 2019 (COVID-19) pandemic has caused a dramatic loss of human life across the globe and presented unparalleled challenges to the world of work. Furthermore, the economic and social disruption caused by the pandemic was catastrophic (Joint statement by ILO, FAO, IFAD, & WHO, 2020). These effects spread across all professions, and academic personnel were not immune to it. The challenges exhibited were in terms of employing flexible teaching approaches, the need to teach courses online, using different platforms to interact with students and colleagues, and innovative ways to carry out research activities ( Superfine, 2020).

Since March 2020, nationally mandated physical distancing led research institutes and universities to adhere to government guidelines in response to the pandemic ( Termini & Traver, 2020). This resulted in unexpected roadblocks for academic personnel with regards to permitted research operations, abiding to physical distancing guidelines in the laboratory, facility closure, decreased laboratory activities, and shifting to remote working ( Termini & Traver, 2020). Further, studies have shown that early career researchers (ECRs), including PhD students and postdoctoral fellows, were affected at the most crucial time in their career development ( Cheng & Song, 2020). Researchers had to switch from working on their current research topic to focusing on COVID-19-based research, while others had to terminate or halt their research work altogether. All these changes impacted the scientist’s ability to conduct research, teach, and their scientific productivity as well. A recent report by Wellcome (2020) highlighted that only 29% of individuals pursuing a research career were secure about their job. This suggested concerns about job security among researchers. Additionally, the findings of the report revealed that poor research culture (like, discrimination, harassment, and unhealthy competition) had a significant impact on researcher’s mental health and the quality of research produced ( Wellcome, 2020). In light of the challenges encountered by the researchers, it is crucial to further assess how the pandemic affected this research environment.

International studies explored how researchers in science, technology, engineering, and mathematics (STEM) fields were coping with changes in routines, funding, productivity, and the like in the wake of the pandemic ( Byrom, 2020; Myers *et al*., 2020). However, the few studies which assessed this impact in India had a very narrow focus such as understanding the impact on a few aspects like funding delays ( Nandita & Joan, 2022), or impact on teaching ( Dar & Lone, 2021). While past studies considered gender as a variable, other factors pertinent to India such as caste, religion, and economic background were not taken into account. Our study aims to incorporate these factors to help understand, in a comprehensive manner, the effect of the COVID-19 pandemic on STEM research scientists and stakeholders (suppliers and funders) across India.

### Primary research discipline and the effect of COVID-19

The COVID-19 pandemic affected researchers in different fields unevenly ( Myers *et al*., 2020). Fields related to the bench sciences, that required physical laboratories, and relied on time-sensitive experiments, such as biochemistry, biological sciences, chemistry, and chemical engineering had large declines in research time when compared to pre-pandemic times. On the other hand, fields that required less equipment such as mathematics, statistics, computer science, and economics reported lower levels of decline in research time ( Myers *et al*., 2020).

Furthermore, Korbel and Stegle (2020) found that one to six months of research work had been lost due to the shutdown of laboratories and that there was a notable difference between dry labs and wet labs. Researchers working in a wet lab reported a greater effect of the pandemic on their work when compared to dry lab researchers ( Korbel & Stegle, 2020). 

### COVID-19 effects on teaching

In addition to difficulties in conducting research, there were other multitude of challenges faced by academic personnel in the domain of teaching. Some of the challenges with online teaching were broadly categorised under accessibility, affordability, flexibility, learning pedagogy, life-long learning, and education policy ( Murgatrotd, 2020, as cited in Pokhrel & Chhetri, 2021). Additionally, many countries lacked reliable internet connection and access to digital sources required for online teaching as well as learning ( Pokhrel & Chhetri, 2021). This made online teaching extremely difficult for both teachers as well as students.

Researchers, who worked in STEM fields in Australia, reported increased challenges in student supervision due to the lack of face-to-face communication, and those with teaching responsibilities had increased teaching workload due to online teaching, thus limiting their research capacity ( EMCR Forum, 2020).

### Difficulty conducting research online

The COVID-19 pandemic changed the way in which we conduct research ( Mitchell, 2021). Individuals who were the most affected were those who lack digital literacy or access to different technologies and research tools required to conduct research online ( Mitchell, 2021). Further, a lack of in-person communication and timeliness led researchers to use online surveys and rating scales to conduct research ( De Man *et al*., 2021), reducing diversity in methodologies.

Clinical trials for stem cell research were gravely impacted by the pandemic as peer review processes could not be worked on without laboratory experiments. In addition, the productivity of stem cell researchers took a hit, especially those amidst a career transition ( Kent *et al*., 2020).

The transition to remote working made it necessary for researchers to have a certain minimum level of digital literacy. Findings from Yazon *et al*.'s (2019) study revealed a strong association between faculty members’ digital literacy and competence to their productivity in research. An increase in understanding, finding, and using information on digital platforms was positively related to faculty members’ ability to conduct research, complete, present, and publish a research article.

In addition to the need for digital literacy among educators, the introduction of virtual laboratories for engineering education involved special training of educators to conduct lab classes. This transformation was received well by both teachers and students ( Kapilan *et al*., 2021).

### Impact of COVID-19 on early career researchers (ECRs)

Scientists at all stages of their careers were impacted by the pandemic; however, early career researchers were significantly vulnerable. A significant impact of the pandemic on ECRs was noted in terms of research productivity, timeline of conducting experiments and research studies, insufficient funding, and interaction with other scientists ( Termini & Traver, 2020). The consequences of these effects were especially severe among the ECRs as it is a crucial period for development and advancement of their career. COVID-19 restrictions led to limitations in collaborative research, informal exchange of ideas, community building, and training offered by the traditional laboratory setting. Furthermore, researchers had insufficient funding due to which they were unable to continue research work and provide scholarly contributions. For some researchers, time-sensitive experiments (e.g., those involving frozen materials) or premature termination of experiments had a negative effect on their studies and also prevented submission of manuscripts due to delays in research work ( Termini & Traver, 2020).

In many cases, open search in the job market was put on hold, due to which ECRs were unable to progress in their careers. Additionally, postdocs who were near the end of their contract had difficulty getting employed and thus, many of them sought employment in non-academic sectors ( Termini & Traver, 2020). Most researchers argued that the pandemic had negatively impacted their career prospects ( Woolston, 2020a). However, another study noted that while students made short-term academic changes that affected their graduation, there were no serious changes to their career plans ( Forakis *et al*., 2020).

A study by Byrom (2020) found that three-fourths of the participants (doctoral students and ECRs from the UK) experienced a negative impact of the lockdown restrictions on their ability to collect data, discuss ideas and findings with colleagues, and disseminate their research findings. Other participants also mentioned that there was a negative impact on data analysis, writing, and working on grant or fellowship applications. Further, there was reduced or no access to the software required for their research work. This decreased ability to work led to stress and worry about researchers’ future plans which resulted in low levels of mental well-being, culminating in mental distress. Additionally, it was found that researchers who had lesser social support networks within and beyond academia tended to struggle with their mental well-being. Administrative burden undertaken by junior researchers due to remote work arrangements contributed to pressure for ECRs ( Matthews *et al*., 2021).

Researchers faced high levels of stress ( Shin & Jung, 2014, as cited in Camerlink *et al*., 2021) and uncertainty with regards to job position ( Castellacci & Vinas-Bardolet, 2021, as cited in Camerlink *et al*., 2021) especially since the onset of the pandemic. It was noted that researchers were facing additional mental health challenges and a reduction in life satisfaction due to the pandemic ( Ammar *et al*., 2020, as cited in Camerlink *et al*., 2021). 

The Australian Academy of Science, Early and Mid-career Researchers (EMCR) Forum conducted a national survey (2020) to understand the impact of COVID-19 on EMCRs in STEM fields in Australia. They found that the pandemic had a significant impact on mental health and productivity of scientists. Researchers perceived a loss of their career prospects and increased anxiety due to uncertain employment situations.

### Gender, race, and impact on research productivity during COVID-19

The stay-at-home orders, lockdowns, and school closures affected scientists, especially those who had to take care of children and elders ( Kowal *et al*., 2020; Myers *et al*., 2020). STEMM (science, technology, engineering, mathematics, and medicine) faculty had to manage their laboratory, transition to remote working, transfer courses to online platforms, continue to be academically productive and also, simultaneously take care of, and home-school their children ( Krukowski *et al*., 2021).

The notion that the lockdown had a differential impact on men and women received considerable recognition ( Muric *et al*., 2021; Yildirim & Eslen-Ziya, 2021). Women academic personnel faced unequal work-life balance challenges during the pandemic, which led to a reduction in the time spent on research hours as compared to men ( Deryugina *et al*., 2021; Myers *et al*., 2020). In a dual-academic relationship, women were more likely to get lesser support at home than men ( King & Frederickson, 2021). Research indicated that women were significantly underrepresented in tenured faculty positions ( Snyder *et al*., 2019, as cited in King & Frederickson, 2021), particularly in STEM fields ( Burrelli, 2008, as cited in Fox, 2001; King & Frederickson, 2021).

In general, productivity in academia is characterised by submitting grants and articles, publication success, as well as other activities, such as peer review and serving on funding panels, which are essential for promotion and tenure ( Krukowski *et al*., 2021). A study by Krukowski *et al*. (2021) found significant changes in productivity before and during the pandemic, with significantly fewer first/corresponding and co-authored articles submitted by women researchers. Further, there were significant decreases in productivity for individuals with children younger than the age of 6 years at home. However, on the other hand, individuals with children between the ages of 6 and 18 years at home, reported significant increase or stable productivity.

Additionally, women’s rate of productivity in last authorship positions declined significantly, suggesting that women were being underrepresented in prestigious, and all other authorship positions. This led to an increased inequality between both genders during the pandemic. Further, there was a significant reduction in women authorships in the first, middle, last, and sole author positions in articles deposited to the arXiv repository, which covers preprints in the fields of physics, maths, statistics, biology, to name a few ( King & Frederickson, 2021). It was also noted that the daily routine of women researchers due to having children was disproportionately affected by the lockdown as compared to men. Thus, on account of the increased domestic burden and child care responsibilities during COVID-19, their integrated impact on career productivity was a threat to tenure and promotion of early career women researchers ( Cardel *et al*., 2020).

Among biodiversity researchers and conservationists in India, COVID-19 affected research, education, communication, networking, and on-field research activities ( Ramvilas *et al*., 2021). In a national study, it was noted that female EMCRs with caring responsibilities, researchers who were early in their career, and researchers working on contract were the groups that were most impacted by the pandemic ( EMCR Forum, 2020).

Apart from gender, an ethnographic study in India had noted that Brahmins and other upper castes dominated in science, medicine, engineering, and academic professions and culturally shaped institutions based on their caste identities ( Thomas, 2020). In a survey conducted by National Institutes of Health (NIH) to understand the impact of the pandemic on scientists belonging to underrepresented racial and ethnic groups, participants reported a decrease in research productivity ( NIH, 2020). A study of scientists in the USA revealed that male researchers without children were the least affected group in terms of productivity during the pandemic as compared to Black mothers, which were the most affected. Racism against black women in academia was also highlighted ( Staniscuaski *et al*., 2021). 

### Institutional and social support

In a study conducted by Ogilvie *et al*. (2020) to understand graduate students’ experiences during the pandemic, most of the respondents mentioned that they received more support from their advisors, professors, and peers rather than from college or university administrators. Additionally, they also reported more support in terms of physical and mental well-being as compared to economic well-being ( Ogilvie *et al*., 2020).

In developing countries such as Bangladesh, it was argued that institutional support during the pandemic was important to fill the academic gap that emerged due to the transition to a virtual education system ( Ullah *et al*., 2021). Institutional support links various stakeholders to resources, expertise, and emotional support allowing navigation through the institution effectively and successfully ( Ullah *et al*., 2021). Ullah *et al*. (2021) assessed the amount of institutional support received in Bangladesh for online education during the pandemic. They found that even though a few universities provided average support for continuing online education, several problems such as lack of software to conduct classes online, lack of training, lack of smartphones, poor internet access, etc. were prevalent.

### Impact of the pandemic on funders and suppliers

In an interview conducted by Nature Communications ( Matthews *et al*., 2021), STEM researchers noted several changes that had occurred in research funding for STEM, and overall in the scientific community. Many funding agencies eased eligibility criteria in order to accommodate students who required funding. Researchers acknowledge that while budget cuts might last longer than the pandemic, philanthropic donations may aid the situation of public universities ( Matthews *et al*., 2021).

The operations and supply chain management were influenced by the COVID-19 pandemic to a large extent ( Lin *et al*., 2020 as cited in Queiroz *et al*., 2020). Disruptions to any of the global supply chains (e.g., closed or partially closed manufacturing units, airports operating with harsh restrictions, shortage of medical equipment and supplies), could lead to the experience of ripple effects by many industries like, medical equipment, consumer good, to name a few ( Dolgui *et al*., 2018; Ivanov, 2020a; Ivanov, 2022, as cited in Queiroz *et al*., 2020). Further, there was an increase in demand for necessary items such as personal protective equipment (PPE), ventilators, and canned foods due to the pandemic. However, because of the various challenges faced by supply, transportation, and manufacturing units, there was a reduction in their capacities. The challenges faced by these units included border closures, lockdown in the markets, interruption in vehicle movement, suspension of international trade, labour shortage, and maintaining physical distancing in manufacturing facilities ( Amankwah-Amoah, 2020; Paul & Chowdhury, 2020, as cited in Chowdhury *et al*., 2021). This substantially affected the suppliers’ ability to deliver products on time ( Ivanov & Das, 2020, as cited in Chowdhury *et al*., 2021). Researchers across the world faced difficulties in securing supplies like gloves, micropipettes, pipette tips, centrifuge tubes, and other laboratory basics leading to an increased demand while the manufacturing and the distribution channels were disrupted ( Woolston, 2021).

The world’s major scientific funders modified their funding policies in response to COVID-19 ( Stoye, 2020). Horizon 2020, a European funding programme for research and innovation, provided researchers with extensions in their funding, and also allowed them to reallocate funds to working remotely and paying salaries of researchers who could not continue with their experiments because of the lockdown. Further, reorientation of the projects to research on COVID-19 was also supported. Other funding institutions such as Cancer Research UK, the Wellcome Trust, US National Institutes of Health (NIH), US National Science Foundation (NSF), and many more provided maximum flexibility and relief to researchers impacted by the pandemic ( Stoye, 2020). The NIH established the COVID-19 supplemental fund to assist affected researchers. They extended the early-stage investigator status and provided significant flexibility in terms of grant money utilisation ( NIH, 2020).

Funding agencies in China, Italy, UK, and USA provided no-cost grant extensions and extended grant deadlines ( Colbert *et al*., 2020). The Canadian Institutes of Health Research (CIHR), a health research investment agency, also implemented gender policy interventions during the COVID-19 funding competition that included extending deadlines and factoring sex/gender into the grant requirements. It was noticed that the CIHR received more applications and awarded a greater proportion of grants to female scientists compared to male scientists. Along with that, many funded studies considered sex and gender in COVID-19 related research ( Witteman *et al*., 2021). 

### Impact on STEM students (or those without a PhD degree)

A study by Gupta *et al*. (2021) reported that most US students' academic path was affected due to the pandemic, while also creating a challenge in completing coursework for degree requirements. Further, they faced difficulty with regards to remote learning, displacement, and loss of opportunities. It was also noted that STEM majors showed concerns with regards to finding internship opportunities, quality of learning, academic performance, and being unprepared for on-site lab and advanced courses.

Another research from the US reported that restrictions on access to resources and facilities along with academic coursework-related challenges led to a delay in graduation by doctoral, masters, and undergraduate students. It was further noted that Hispanic and Black undergraduates were more likely than Asians and Whites to delay graduation (Report 1; Saw *et al*., 2020a). It was also observed that STEM female faculty and students reported facing more problems adapting to remote learning and technological issues compared to their male colleagues and peers (Report 2; Saw *et al.*, 2020b). Furthermore, it was noted that PhD students in Brazil belonging to a minority ethnic group were more likely to be financially disadvantaged compared to white students ( Woolston, 2020b).

### Positive outcomes of the pandemic

Ranganathan *et al*.'s (2021) study on cancer care during the pandemic also highlighted the increase in value-based health care which involved focusing on a patient’s outcome-based treatment wherein unnecessary tests were avoided and the provider was also monetarily compensated based on the patient's health outcome. This included initiatives such as ‘Choosing Wisely’ for cancer patients, in addition to telephonic consultations. COVID-19 research illustrated efficient ways of doing clinical cancer research that included reduced imaging. This was learnt from large scale practice-defining trials resulting in the modification of existing cancer trial protocols. 

COVID-19 also had a significant impact on scientific communication, collaboration, and training. Video conferencing gained importance in terms of meetings, journal clubs, and communication with collaborators. In a study conducted among life science scientists, more than half of the participants suggested that their communication with mentors or supervisors had not changed and a few participants also noted an increase in communication. This indicated that video conferencing was effective in communication and mentoring during the pandemic ( Korbel & Stegle, 2020).

It was also noted that e-conferencing among life science scientists was becoming an important format for scientific meetings. During the lockdown, the adoption of e-learning software by life science trainees based in wet labs increased. The trainees wanted to expand their skill set like, learning new programming languages ( Korbel & Stegle, 2020). Further, scientists spent more time in data analysis, manuscript or thesis writing, and developing grant applications. Some scientists also indicated shifting their research activities to contribute to COVID-19 related research ( Korbel & Stegle, 2020). In sum, even though the pandemic had substantial effects associated with stress and work interruptions among scientists, new ways to cooperate, exchange ideas, and learn via electronic means were some of the positive outcomes of the pandemic ( Korbel & Stegle, 2020).

Vast literature emphasised the scope of the impact of COVID-19 pandemic on STEM researchers all over the world. In particular, the pandemic had a significant impact on ECRs, who faced a barrier in the progression of their career, as well as women scientists who were unable to work to their full potential due to household or childcare responsibilities. While most of these challenges were already faced by many researchers prior to the pandemic, these issues were heightened during the pandemic. However, not many of these studies focused on the pandemic’s influence on Indian scientists. Therefore, the current study aims to understand the effect of gender, caste, childcare responsibilities, primary research discipline, transition to online working/ teaching, contracting COVID-19, funding opportunities, and institutional and social support received on scientific productivity, mental health and future career prospects among researchers in India.

### Research questions

In the context of emerging strands of literature on the impact of COVID-19 on STEM research, the current study posits the following research questions in the Indian context:

RQ1: What impacts the ability to continue one’s research during the COVID-19 pandemic?

RQ2: What impacts one’s ability to continue to teach during the COVID-19 pandemic?

RQ3: What impacts researcher’s scientific productivity during the COVID-19 pandemic?

RQ4: What impacts mental health among STEM scientists during the COVID-19 pandemic?

RQ5: What has an impact on a STEM scientist’s decision to return to academia, after leaving academia during the COVID-19 pandemic?

RQ6: What has an impact on a STEM scientist’s plan to continue a career in STEM even if they are thinking about leaving academia?

RQ7: What was the differential impact of the pandemic among ECRs, Heads of Institutes, suppliers and funders?

RQ8: What were some of the reasons behind planning to leave academia?

RQ9: What were the reasons and effects of leaving academia?

RQ10: Were there any actionable policy recommendations that arise from various challenges faced by scientists during the pandemic?

## Methods

### Ethical considerations

The study was approved by the Monk Prayogshala Institutional Review Board (FWA-recognized) on 5 ^th^ July 2021 (#065-021). Written informed consent from survey participants and audio-recorded consent from interview participants for publication of unidentifiable participant responses was obtained.

### Design

The current study employed a mixed method design using both quantitative (survey) and qualitative (interview) methods to collect data from researchers and stakeholders in STEM. To make the survey ^1^ more accessible to participants and to recruit a representative sample, the survey was made available in ten Indian languages (Hindi: 75, Marathi: 24, Tamil: 13, Kannada: 6, Telugu: 1, Bengali: 18, Gujarati: 7, Malayalam: 11, Oriya: 3, and Assamese: 4) along with English (n = 912). 

### Participants

Participants were recruited via targeted emails to Institute and Department heads, networks of India Alliance and Dr. Subramanyam, comprehensive database of Central Institutes in India, and snowball sampling through social media campaigns. The sample size for the study was stipulated by the funding agency (DBT/Wellcome Trust India Alliance). The study included participants from India who were 18 years and above, and those studying or working in a STEM-related field. Data from participants were excluded from the analysis if the participant did not consent to participate in the study, the progress for the survey was either 0 or 1, and those younger than the age of 18 years.

Heads of Institutes, suppliers of scientific material, and funders/donors for the interview were recruited using purposive sampling. Contact information for all potential respondents was collected from websites of research institutes, organisations that work in STEM disciplines, government research institutes, universities, companies that supply scientific equipment and funding agencies working in India. Another method of recruiting respondents included using the India Alliance's network of fellows who work in various institutes across the country. The fellows were contacted and asked if they could put the authors in touch with their respective heads of institutes to be interviewed. The study was conducted to obtain representation from all regions of India and from researchers working in government research laboratories, universities, private institutes, and colleges.

### Measures

***Survey*.** The survey form was designed and circulated online via Qualtrics. It was a self-developed tool that included questions related to participant’s socio-demographics, the effects of COVID-19 on research, funding, scientific productivity, teaching, institutional/social support, mental health, and details on COVID-19 information. Further, the survey also included questions for researchers who had left/were thinking of leaving academia. Double-barrelled questions were avoided in the survey. Furthermore, display and skip logic functions were used in the survey so that participants did not have to respond to questions that were not applicable to them thus reducing fatigue.

***Interview*.** These were scheduled with the heads of institutes, suppliers of scientific materials, funders/donors, ECRs, people who were thinking about leaving academia, and those who had already left academia based on mutual convenience. The interview guides included questions based on COVID-19 effects on the institute, funding, scientific productivity, teaching, and social support. The semi-structured interviews were conducted by Vedika Inamdar, a female research author at the department of sociology at Monk Prayogshala, India. The researcher has a Master of Arts (M.A.) degree and has 3 years of training and experience as a qualitative researcher.

The semi-structured interview schedule was pilot tested. Each online interview typically lasted between 45 to 60 minutes and was recorded (audio and/or visual) on the Zoom Meeting Platform with prior consent of the interviewee for transcription at a later stage. No repeat interviews were conducted for any of the participants. Furthermore, leading questions were not asked in the interviews to avoid biased responses.

The interview schedule and questionnaire can be found as *Extended data* ( Mehta *et al*., 2022).

### Procedure

The survey form included quantitative as well as a few qualitative questions for a detailed understanding on individuals experiences during the pandemic. At the end of the survey, participants were debriefed about the study and were provided with the option of entering their email ID to receive a compensation of INR 100 and a certificate of participation from India Alliance and Monk Prayogshala for taking part in the study. All semi-structured interviews were conducted online, using Zoom video-conferencing software and audio-recorded for transcription at a later stage. At the end of the interview, participants were debriefed about the study and were provided with a compensation of INR 1000 and a certificate of participation from India Alliance and Monk Prayogshala for taking part in the study.

### Data analysis

Quantitative data were analysed using RStudio software version 1.4.1717 ( RStudio team, 2021). The factor structure and internal consistency reliability of the self-developed survey questions were assessed using confirmatory factor analysis (CFA) and Cronbach’s alpha. One-factor CFA models were computed to understand whether the tested variables represented the specified construct. Furthermore, Cronbach's alpha tested to understand the inter-relatedness of the items in a scale. Based on these metrics, indices for digital literacy, core research issues, university support, social support, and mental health were developed. Next, zero-order correlations were assessed based on which regression analysis was computed to assess the proposed research questions. To corroborate these findings, sentiment and content analysis was computed on the descriptive responses provided by the participants. Additionally, interview responses were analysed using thematic analysis. This analysis was coded by two qualitative researchers using the NVivo 20 software (released in March 2020).

## Results

### Quantitative results

The participants of this study were STEM ECRs (within 10 years of receiving PhD), senior postdoctoral fellows, researchers with their own labs/groups with less than 10 years of research experience, those having a graduate/postgraduate degree, heads of institutes, suppliers of scientific materials, and funders/donors. A total of 1074 participants took part in the online survey. Participants not meeting the inclusion criteria were excluded; thus, a total of 618 participants were included in the analysis ( Mehta *et al*., 2022). Specifically, participants who completed the survey in less than 90 seconds (n = 351), those with a progress of 0/1 (n = 81), individuals who did not consent to participate (n = 2), and participants younger than 18 years, those with variables having extreme values, and participants not falling into the criteria for ECRs (especially for those who had their doctoral degree; n = 22) were excluded from analysis. Finally, the dataset was divided into two groups, one for those who had completed their doctoral or postdoctoral training (N = 300) and another for those who have only completed their post-graduation or graduation (N = 318). The sample size reduced further for certain variables owing to missing data (refer to the descriptive tables for more detail).

***Participants having a doctoral or a postdoctoral degree***

**Descriptive statistics.** The dataset included a total of 150 men, and 141 women (6 participants preferred not to disclose their gender) having a mean age of 39.43 years (SD = 7.46). Out of the total number of participants, 162 individuals had a doctorate (MD or PhD) degree and 138 individuals had completed their postdoctoral training. Additionally, 149 of the total participants belonged to a dominant caste group (Brahmin, Kshatriya, Vaishya, and other upper castes) whereas, 36 participants belonged to an underprivileged caste group (Scheduled Caste, Scheduled Tribe, Other Backward Class, and other lower castes), while the remaining participants did not disclose their caste details. For more details, refer to Table 1 and Table 2 in the Appendix.

**Reliability and validity.** Indices for variables such as digital literacy, core research issues, university support, social support, and mental health were developed. Cronbach’s alpha and confirmatory factor analysis using the MLR (robust maximum likelihood) method of estimation was computed in order to evaluate the psychometric properties of the indices. Additionally, since digital literacy, core research issues, and social support indices were found to be non-normal (see Table 3), a DWLS (diagonally weighted least squares) method of estimation was also computed to assess index validity. For the factor models, fit was measured by evaluating the comparative fit index (CFI), the Tucker-Lewis index (TLI), the root mean square error of approximation (RMSEA), and standardised root mean square residual (SRMR), in order to determine optimal fit (see Table 4). According to the widely used criteria, a cut-off value of ≥0.95 for CFI and TLI, ≤0.06 for RMSEA, and ≤0.08 for SRMR indicate a good model fit ^2^ ( Groskurth *et al.*, 2021; Hu & Bentler, 1999).

**Table 3.** Shapiro-Wilk test of normality.

Indices	W	p-value
Digital Literacy	0.68	0.000
Core research issues	0.98	0.021
University support	0.99	0.354
Social support	0.98	0.014
Mental health	0.99	0.713

*Note.* W = Shapiro–Wilk test statistic

**Table 4.** One-factor confirmatory factor analysis using robust maximum likelihood (MLR) and diagonally weighted least squares (DWLS) methods.

Indices	No. of items	N	Estimation	CFI	TLI	RMSEA	SRMR
Digital Literacy	6	160	MLR	0.827	0.712	0.343	0.062
			DWLS	1.00	1.042	0.00	0.062
Core research issues	8	133	MLR	0.846	0.784	0.129	0.078
			DWLS	0.988	0.983	0.043	0.077
University support	10	121	MLR	0.691	0.603	0.183	0.113
			DWLS	NA	NA	NA	NA
Social support	5	163	MLR	0.727	0.454	0.303	0.139
			DWLS	0.851	0.703	0.207	0.133
Mental health	4	168	MLR	1.00	1.004	0.00	0.022
			DWLS	NA	NA	NA	NA

*Note.* CFI = Comparative Fit Index, TLI = Tucker Lewis Index, RMSEA = Root Mean Square Error of Approximation, SRMR = Standardized Root Mean Square Residual.

For the dataset involving individuals who had completed their PhD or postdoctoral degree, it was noted that the digital literacy index ( ***α*** = 0.93), the core research issues index ( ***α*** = 0.80), university support index ( ***α*** = 0.84), social support index ( ***α*** = 0.72), and the mental health index ( ***α*** = 0.70,) had a good internal consistency reliability. ^3^

The core research issues index involved items related to difficulty in discussing research with colleagues, difficulty in data collection, difficulty in dissemination, methodological challenges, lab staff being asked to leave, decrease in lab staff, staff leaving affecting performance, and staff unable to continue research work on campus. The digital literacy index measured the participants’ ability to access email, virtually access bank accounts, use digital technologies, video conferencing, online file sharing, and learning new technology without the help of a third party.

University support index included the extent of physical, mental, material, and economic support received from university professors and administrators. Furthermore, support received from the university in terms of resources, flexibility in work hours, training, monetary assistance, and financial guidance were also measured. Support received from family, relatives, and peers in terms of physical, mental, material, and economic well-being were included in the social support measure. Mental health index included items related to overall mental health, work-life balance, amount of stress and happiness one experienced.

**Correlations.** A Pearson’s correlation coefficient was computed to understand the relationship between the variables (see Table 5). It was noted that if the number of people residing in a household along with those below the age of 18 years increased, one’s access to independent workspace reduced. Additionally, a negative impact on teaching was positively correlated to difficulty in migrating to online teaching.

**Table 5.** Correlation matrix (participants with a doctorate/post doctorate degree).

Variable	*1*	*2*	*3*	*4*	*5*	*6*	*7*	*8*	*9*	*10*
1. Age										
2. Receive PhD/postdoc degree	-.83 **									
3. People residing in household	-0.06	-0.03								
4. People residing in household below 18yrs	0.01	-0.03	.43 **							
5. People residing in household above 60yrs	-.13 *	0.05	.45 **	.20 **						
6. Caregivers in household	-.19 **	0.09	.21 **	.20 **	.25 **					
7. Access to independent workspace	0.08	-0.01	-.14 *	-.15 *	-0.02	0.08				
8. Depend on lab	-0.05	.17 *	0.09	-0.01	-0.01	0.01	-0.06			
9. Human participants	0.09	-0.1	0	0.08	-0.03	-0.05	-0.01	.22 **		
10. Remote working	.14 *	-0.09	-0.04	-0.07	0.02	-0.09	.34 **	-.29 **	-.13 *	
11. Stable internet connection	0	0.01	-0.07	-0.02	0.02	0.07	.44 **	-0.06	-0.01	.35 **
12. Disruption in supplies	0.04	0.08	0	-0.01	-0.06	0.01	-.17 *	.57 **	0.11	-.30 **
13. Core research issues-total	0.07	-0.02	-0.01	-0.01	-0.12	0.04	-.28 **	.30 **	0.1	-0.1
14. PhD degree delay	-0.02	0.08	-0.11	0.01	-.19 *	-0.01	-.19 *	0.09	-0.08	-0.14
15. Postdoc training delay	-0.12	.19 *	-0.05	-0.01	-0.09	0.05	-.29 **	0.15	0.02	-.19 *
16. Administration time	0.11	-0.12	0.05	0.01	-0.1	-.18 *	-0.01	0.02	0.01	.18 *
17. Professional development	-0.01	0.01	0.13	-0.09	0.06	-0.03	0.01	-0.03	-.16 *	0.15
18. Digital literacy-total	0	-0.05	-0.06	-.19 *	-0.04	-0.01	.16 *	0.08	0.05	0.06
19. Difficulty receiving grant	-0.1	0.06	-0.04	0.05	0.03	0.07	-0.06	0.08	-0.06	0.11
20. Personal financial stability	-.17 *	.18 *	.17 *	0.15	0.09	-0.03	-.29 **	.16 *	0.15	-0.1
21. Household financial stability	-0.1	0.14	.19 *	0.1	0.06	-0.01	-.22 **	.16 *	0.1	-0.06
22. Scientific productivity	-0.01	0.14	0.04	0.02	0.04	0.05	-0.04	.31 **	.21 **	0
23. Impact on supervisory role	0.09	0	-0.13	-0.03	-.23 *	0.07	-.28 **	-0.03	0.13	-.27 *
24. Migration to online teaching	0.06	-0.09	-0.1	-0.02	-0.08	0.07	-0.17	-0.06	0.09	-0.01
25. Negative impact on teaching	0.09	-0.05	0.05	0.03	-0.04	0.13	-0.08	-0.01	0.03	0.03
26. University support-total	0.03	-0.15	0.09	0.05	0.04	0.12	0.1	-.19 *	-0.02	.20 *
27. Social support-total	-0.03	-0.01	-0.07	-0.09	0.03	0.03	.36 **	0.06	-0.08	0.1
28. Mental health-total	0.04	-0.09	0.01	0.06	-0.05	0.05	.30 **	-0.07	-0.14	0.14

Variable	*11*	*12*	*13*	*14*	*15*	*16*	*17*	*18*	*19*	*20*
11. Stable internet connection										
12. Disruption in supplies	-.19 **									
13. Core research issues-total	-.23 **	.40 **								
14. PhD degree delay	-.18 *	.21 **	.32 **							
15. Postdoc training delay	-0.13	.25 **	.40 **	.68 **						
16. Administration time	0.08	0.06	0	-0.02	-0.05					
17. Professional development	0.06	-0.05	-0.05	-0.03	0.05	.48 **				
18. Digital literacy-total	.33 **	0	-0.13	-0.02	-0.04	.22 **	.17 *			
19. Difficulty receiving grant	-0.06	.29 **	0.12	0.05	0.08	0.01	-0.03	-.18 *		
20. Personal financial stability	-.30 **	.26 **	.19 *	0.05	0.13	-0.04	-0.02	-.22 **	.26 **	
21. Household financial stability	-.37 **	.26 **	.21 **	0.02	0.13	-0.02	0	-.23 **	0.15	.83 **
22. Scientific productivity	-0.08	.32 **	.36 **	0.17	.25 **	0.12	.20 **	0.07	.21 *	.29 **
23. Impact on supervisory role	-.30 **	.34 **	.46 **	.46 **	.36 **	-0.1	-.24 *	-0.08	-0.02	0.12
24. Migration to online teaching	-.20 *	0	0.04	.33 **	0.11	-0.03	-0.07	0	-0.01	0.04
25. Negative impact on teaching	-0.17	0.07	.26 **	.30 **	.23 *	0.08	-0.01	-0.08	0.01	0.08
26. University support-total	.27 **	-.20 *	-.20 *	-.25 **	-.23 *	.18 *	-0.02	-0.05	-.30 **	-.24 **
27. Social support-total	.22 **	-0.11	-.29 **	-0.08	-0.15	0.11	0.14	.18 *	-0.04	-.18 *
28. Mental health-total	.24 **	-.20 *	-.34 **	-.30 **	-.29 **	-0.01	0.03	0.11	-.29 **	-.26 **

Variable			*21*	*22*	*23*	*24*	*25*	*26*	*27*	*28*
21. Household financial stability										
22. Scientific productivity			.23 **							
23. Impact on supervisory role			0.12	0.19						
24. Migration to online teaching			0.12	0.05	.46 **					
25. Negative impact on teaching			0.12	0.13	.57 **	.47 **				
26. University support-total			-0.15	-.19 *	-.30 **	-0.07	-0.04			
27. Social support-total			-.19 *	-0.04	-.29 **	-0.18	-.20 *	.31 **		
28. Mental health-total			-0.16	-.34 **	-.37 **	-.22 *	-0.11	.41 **	.41 **	

*Note.* * p < .05. ** p < .01.

To further summarise the findings, greater social support was correlated with lower core research issues, a decrease in impact on supervisory role, and a decrease of negative impact on teaching. On the other hand, decrease in university support is correlated to an increase in disruption of lab supplies, core research issues, delay in PhD degree, delay in postdoc completion, disruption in receiving a grant or fellowship, personal financial instability, and impact on supervisory role. In terms of scientific productivity, an adverse change in productivity was related to an increased reliance on a lab to conduct research, dependency on interaction with human participants, disruption in lab supplies, core research issues, and a lower university support.

Finally, better mental health was correlated with increase in access to an independent workspace, better stable internet connection, and greater social and university support. Additionally, better mental health was also related to decrease in disruption of lab supplies, reduced difficulty receiving a grant, greater personal financial security, no change in productivity, lower impact on supervisory role, decreased difficulty migrating to online teaching, and a reduction in students' PhD degrees delay and postdoctoral scholars’ training delay.

**Regression analysis ^4^.** Based on significant correlations between variables, multiple regression models were computed using pairwise deletion (lavaan; Rosseel, 2012) to answer each above-mentioned research question (see Table 6). Additionally, regression analysis was also performed on disaggregated datasets based on gender (males and females) and caste (dominant and underprivileged caste). A *post hoc* power analysis using G*Power 3.1 ( Faul *et al*., 2009; Faul *et al*., 2007, RRID:SCR_013726) was computed for all the models having at least one significant predictor. It was observed that the models had a high power ranging from 0.95- 1.00 ( *α* = 0.05) for the differing effect size, sample size, and number of predictors for each model. Regression results allow us to examine specific hypotheses related to certain variables, while controlling for other confounding variables. In this way, the results focus on the statistically significant (or otherwise) association or effect between the explanatory variable and the outcome(s) of interest.

**Table 6.** Multiple regression model estimates for each research question.

Research Question	Full Sample		Men		Women		Dominant caste	
	*N*	R ^2^	*N*	R ^2^	*N*	R ^2^	*N*	R ^2^
RQ1- What impacts the ability to continue one’s research during the COVID-19 pandemic?-Core research issues	233	0.158	117	0.27	113	0.083	127	0.158
RQ1- What impacts the ability to continue one’s research during the COVID-19 pandemic?-Logistic issues (Disruption in supply)	248	0.15	122	0.18	122	0.134	135	0.147
RQ1- What impacts the ability to continue one’s research during the COVID-19 pandemic?-Peripheral issues (Professional development)	172	0.031	88	0.055	83	0.008	90	0.02
RQ2- What impacts one’s ability to continue to teach during the COVID-19 pandemic?- Impact on supervisory role	248	0.28	122	0.339	122	0.414	135	0.407
RQ2- What impacts one’s ability to continue to teach during the COVID-19 pandemic?- Difficulty migrating to online teaching	245	0.069	120	0.094	121	0.068	123	0.271
RQ2- What impacts one’s ability to continue to teach during the COVID-19 pandemic?-Negative impact on teaching	245	0.057	120	0.074	121	0.049	133	0.106
RQ3- What impacts researcher’s scientific productivity during the COVID-19 pandemic?	248	0.274	122	0.352	122	0.276	135	0.304
RQ4- What impacts mental health among STEM scientists during the COVID-19 pandemic?	248	0.395	122	0.388	122	0.468	135	0.393

**Figure 1.**
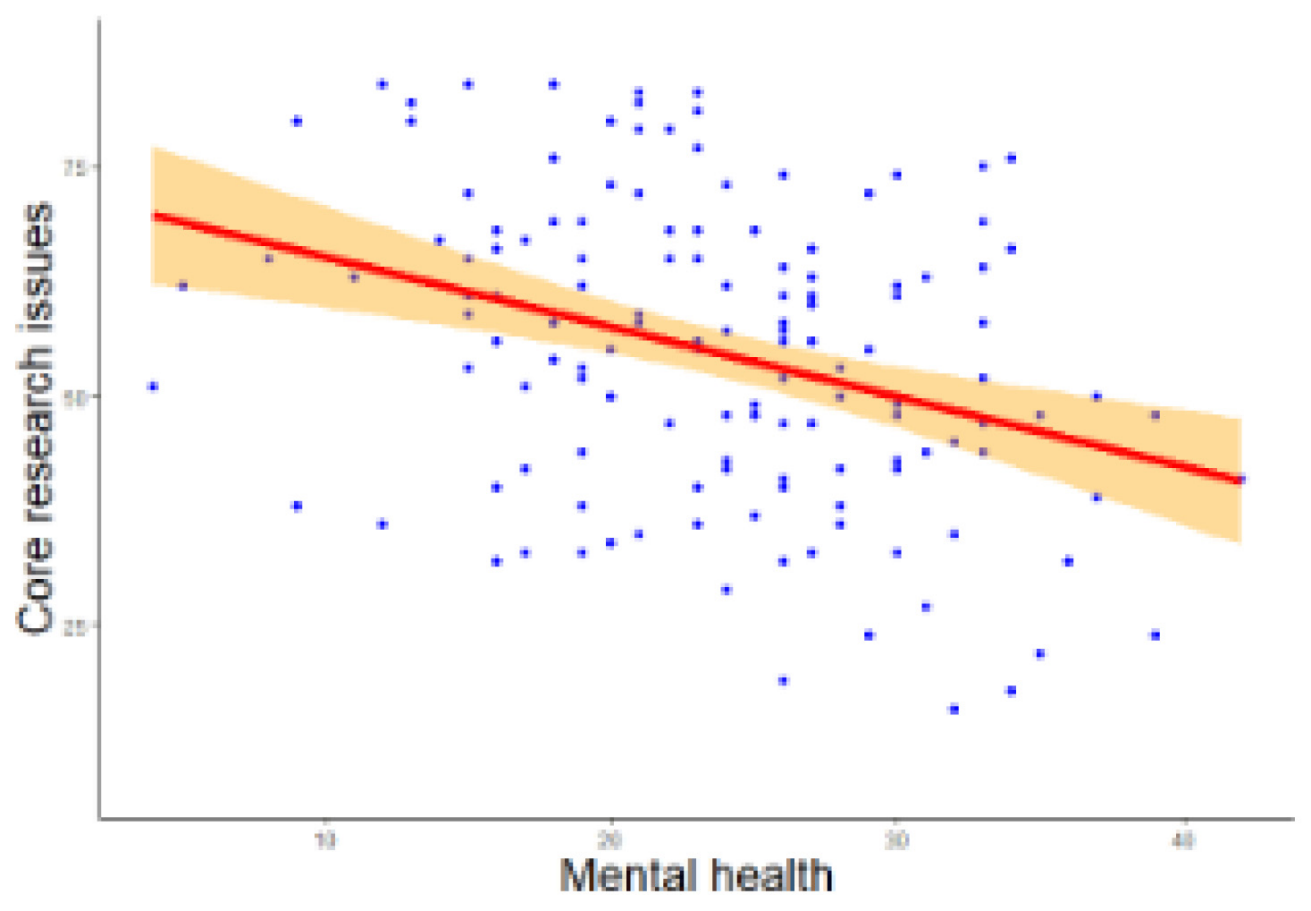
Regression analysis of mental health on core research issues. *Note.* The figure shows a negative relationship between mental health and core research issues.

The results ^5^ ( Figure 1) showed that lower mental health significantly predicted a greater number of core research issues ( *β* = -0.546, z =-2.807, p = 0.005). Furthermore, greater difficulty in receiving a grant, significantly predicted a greater disruption in lab supplies ( *β* = 0.18, z = 2.345, p (p-value) = 0.019), and a higher digital literacy significantly predicted an increase in the number of working hours in terms of professional development ( *β* = 0.034, z = 1.959, p = 0.050). This suggests that mental health, difficulty receiving a grant, and digital literacy had a significant impact on one’s ability to continue one’s research during the COVID-19 pandemic (RQ1).

It was observed ( Figure 2a) that greater disruption in procuring lab supplies had a significantly higher impact on an individual’s supervisory role ( *β* = 0.254, z = 2.051, p = 0.040). Thus, this might be one of the reasons that affected one’s ability to continue to teach during the COVID-19 pandemic (RQ2). Note that no statistically significant relationship was observed between disruption in lab supply and other aspects of online teaching (e.g., migration to online teaching). Further, greater core research issues predicted an adverse change in researcher’s scientific productivity during the pandemic ( *β* = 0.024, z = 2.136, p = 0.033; RQ3). Finally, it was noted that STEM scientists’ better mental health (RQ4) was significantly predicted by a lesser difficulty in receiving a grant ( *β* = -0.343, z = -2.302, p = 0.021, Figure 2b), a smaller change in scientific productivity ( *β* = -0.707, z = -2.602, p = 0.009), higher university support ( *β* = 0.069, z = 2.070, p = 0.038, Figure 2c), and higher social support ( *β* = 0.189, z = 3.963, p = 0.00).

**Figure 2a.**
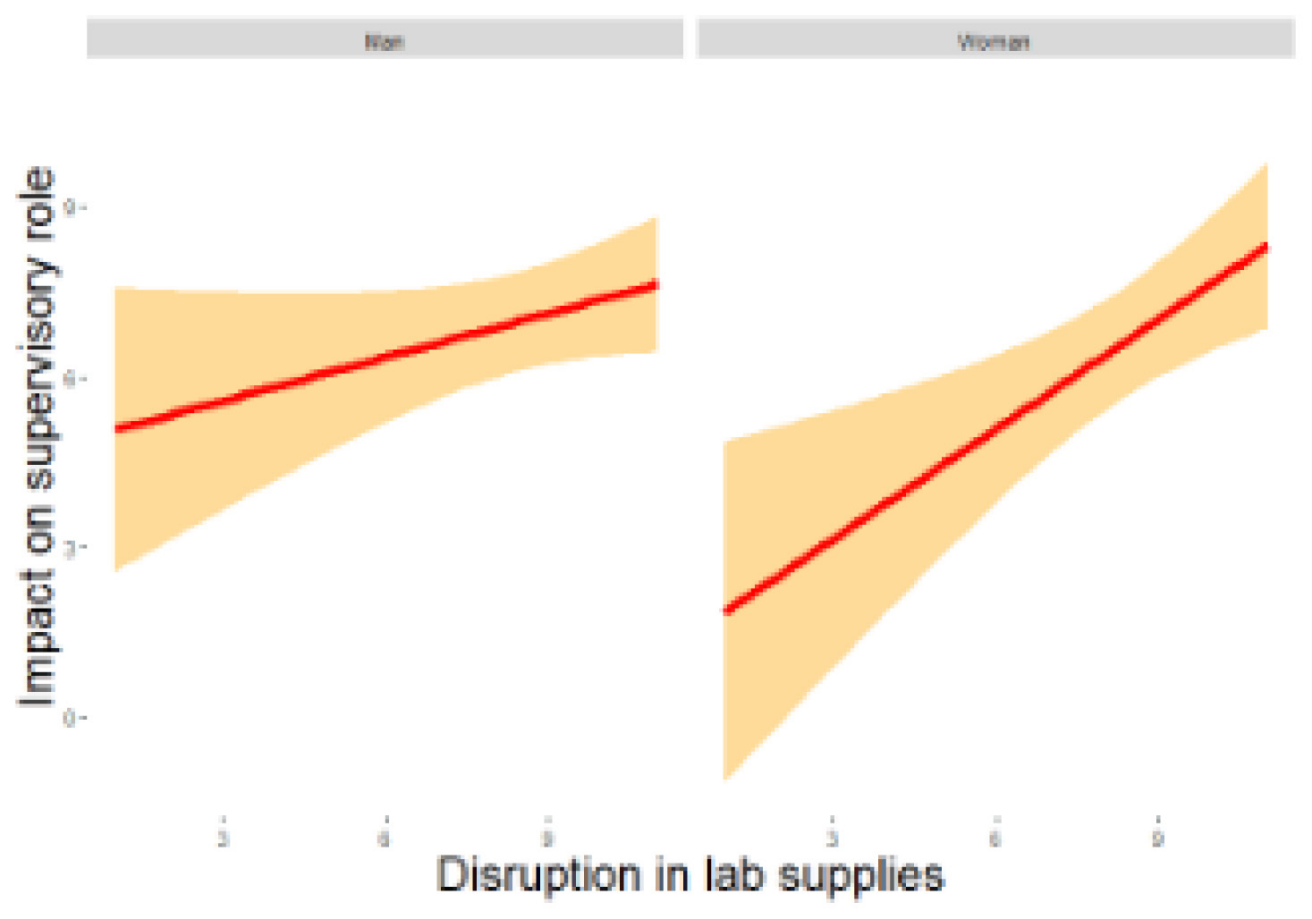
Regression analysis for men and women of lab supplies disruption on supervisory role. *Note.* The figure shows a positive relation between disruption of supplies and impact on supervisory role which was stronger and significant for women as compared to men.

**Figure 2b.**
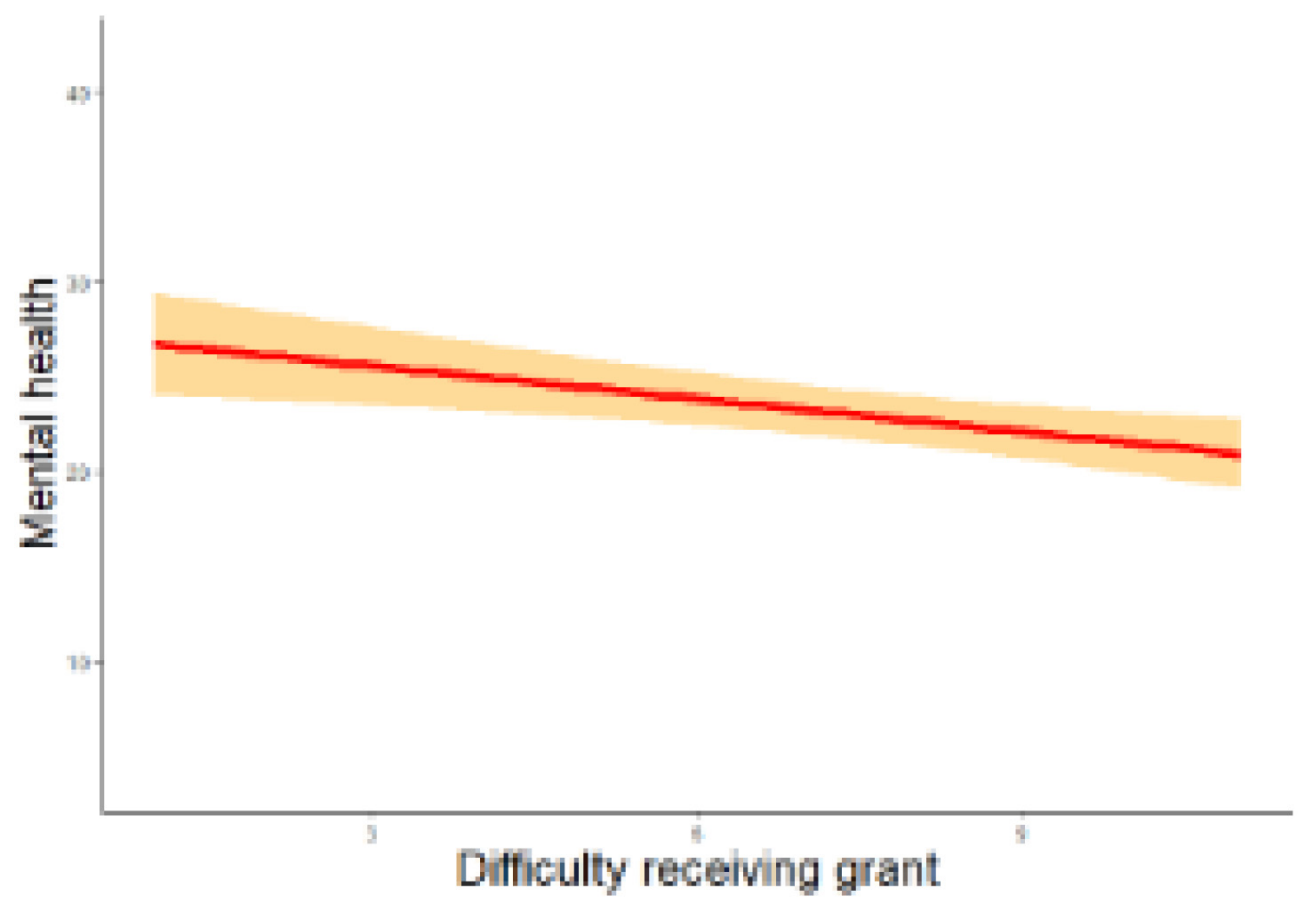
Regression analysis of difficulty of receiving a grant and mental health. *Note.* The figure shows a weak negative relation between difficulty receiving a grant and impact on mental health.

**Figure 2c.**
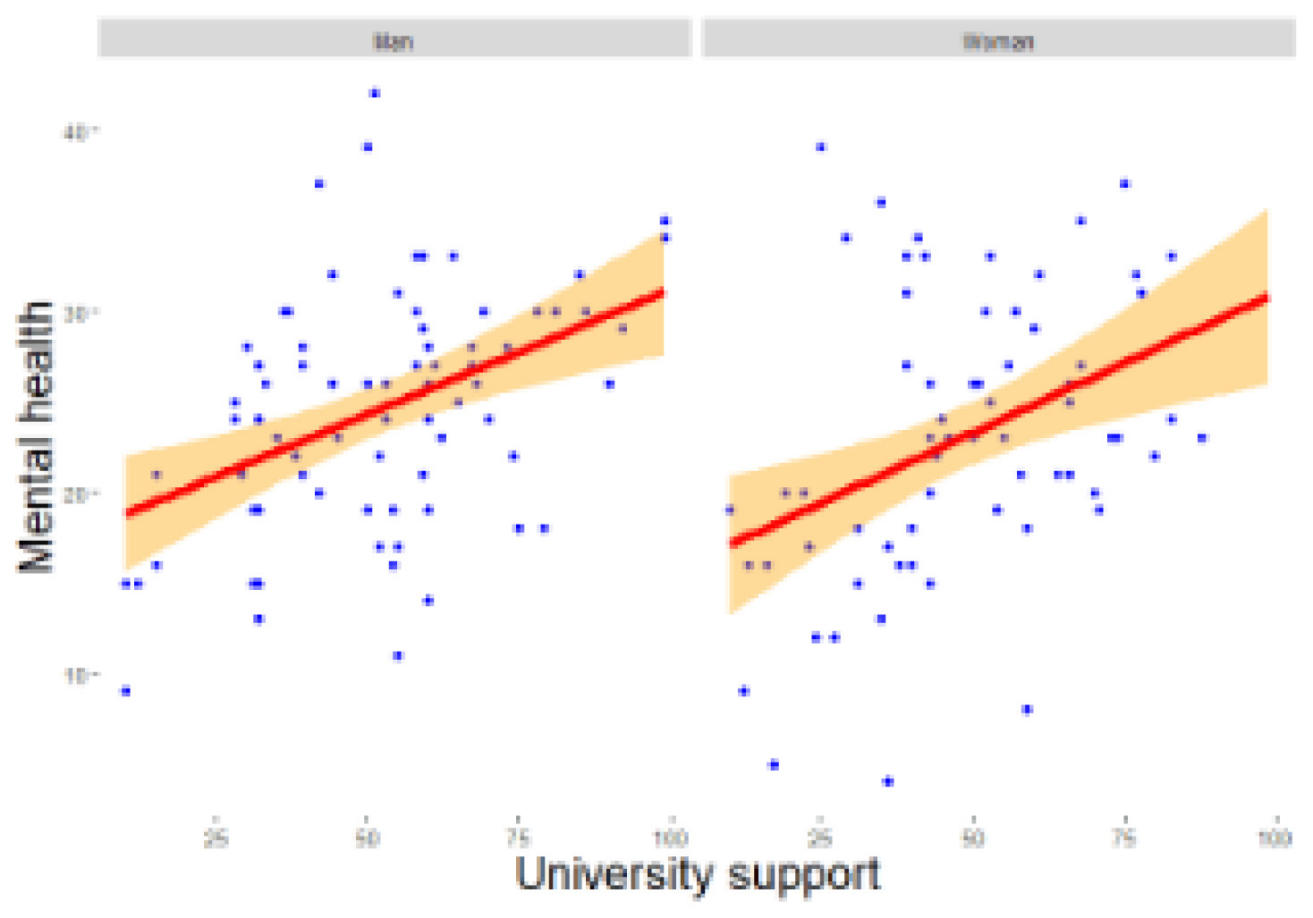
Regression analysis for men and women of university support on mental health. *Note.* The figure shows a stronger and significant positive relationship between university support and mental health among men as compared to women.

For men, it was found that greater core research issues were significantly predicted by lower mental health ( *β* = -0.58, z = -2.152, p = 0.031), and higher the difficulty in receiving a grant predicted a greater disruption in procuring lab supplies ( *β* = 0.252, z = 2.058, p = 0.040). This suggests that mental health and difficulty receiving a grant were major aspects affecting men’s inability to continue research during the pandemic (RQ1). Furthermore, higher the research dependency on interactions with human participants ( *β* = 0.175, z = 2.290, p = 0.022) and greater core research issues ( *β* = 0.039, z = 2.406, p = 0.016) significantly predict adverse changes in scientific productivity for men (RQ3). Higher university support ( *β* = 0.072, z = 2.151, p = 0.031) and social support ( *β* = 0.127, z = 2.015, p = 0.044) predicted a better mental health among men (RQ4).

For women, on the other hand, it was noted that lower mental health significantly predicted higher core research issues ( *β* = -0.547, z = -1.995, p = 0.046) thus, impacting their ability to continue research during the pandemic (RQ1). Additionally, a higher disruption in procuring lab supplies predicted a greater impact on their supervisory role for PhD students ( *β* = 0.402, z = 2.126, p = 0.033), and a lower mental health predicted a greater the difficulty to migrate to online teaching ( *β* = -0.091, z = -1.956, p = 0.050) consequently affecting female’s ability to continue to teach (RQ2). Adverse change in scientific productivity (RQ3) was predicted by greater personal financial instability ( *β* = 0.252, z = 2.850, p = 0.004) and lower mental health ( *β* = -0.081, z = -2.042, p = 0.041). Greater difficulty receiving a grant ( *β* = -0.531, z = -2.508, p = 0.012), adverse change in productivity ( *β* = -0.977, z = -2.929, p = 0.003), and lower social support ( *β* = 0.220, z = 3.378, p = 0.001) significantly predicted lower mental health for women (RQ4).

For dominant castes, who form a majority in our sample, it was observed that being able to manage switching to remote working ( *β* = -0.396, z = -2.876, p = 0.004), better stability in internet connection to work remotely ( *β* = -0.387, z = -2.198, p = 0.028), and lesser disruption in lab supplies ( *β* = 0.285, z = 2.057, p = 0.040) had a lower impact on one’s supervisory role (RQ2). A better stability in internet connection to work remotely ( *β* = -0.608, z = -3.888, p = 0.00) and better mental health ( *β* = -0.103, z = -2.069, p = 0.039) significantly predicted a lower difficulty to migrate to online teaching (RQ2). Further, a higher dependency of working in a physical lab predicted an adverse change in scientific productivity (RQ3; *β* = 0.242, z = 2.168, p = 0.030). It was also noted that greater social support predicted better mental health ( *β* = 0.179, z = 2.328, p = 0.020) among the dominant caste group (RQ4). Note that these are not relative to the underprivileged caste group as there was insufficient data on underprivileged caste group members in the survey.

The underprivileged caste group had a very small sample size (n = 36); hence, the correlations potentially show spurious relationships that might lead to inaccurate inferences, and as a result, are not reported here.

For those who had left academia (RQ5; N = 23) or were thinking about leaving academia (RQ6; N= 24), due to a small sample size, statistically robust and reliable results were not obtained. Hence, qualitative data was used to gauge a scientist’s reasons for leaving or considering leaving academia. This is discussed in the following section.

***Participants having a graduate or a postgraduate degree (i.e., not a PhD)***

**Descriptive statistics.** A total of 175 individuals identified as men, 134 individuals identified as women, and 2 individuals identified as non-binary/transgender (4 participants prefered not to respond). The sample had a mean age of 29.34 years (SD= 8.26) and 177 of the total participants belonged to a dominant caste group (Brahmin, Kshatriya, Vaishya, and other upper castes) whereas, 55 participants belonged to an underprivileged caste group (Scheduled Caste, Scheduled Tribe, Other Backward Class, and other lower castes). For more details, refer to Table 7 and Table 8 in the Appendix.

**Reliability and validity.** Internal consistency reliability and CFA using MLR method of estimation was computed to evaluate their psychometric properties of the indices. Since, the data for all the indices was not normal (see Table 9), DWLS estimation was also used to evaluate the validity of the indices (see Table 10). For the dataset involving individuals who had completed their graduate or postgraduate degree, it was noted that the digital literacy index ( ***α*** = 0.90, robust CFI= 0.980), the core research issues index ( ***α*** = 0.74, robust CFI= 0.986), university support index ( ***α*** = 0.89, robust CFI= 0.818), social support index ( ***α*** = 0.83, robust CFI= 0.787), and the mental health index ( ***α*** = 0.76, robust CFI= 1.00) had a good internal consistency reliability and an adequate model fit¹ ( Groskurth *et al*., 2021).

**Table 9.** Shapiro-Wilk test of normality (participants with a graduate/postgraduate degree).

Indices	W	p-value
Digital Literacy	0.92	1.96E-07
Core research issues	0.98	0.002
University support	0.96	0.00075
Social support	0.98	0.002
Mental health	0.98	0.025

*Note.* W = Shapiro–Wilk test statistic.

**Table 10.** One-factor confirmatory factor analysis using robust maximum likelihood (MLR) and diagonally weighted least squares (DWLS) methods (participants with a graduate/postgraduate degree).

Indices	No. of items	N	Estimation	CFI	TLI	RMSEA	SRMR
Digital Literacy	6	149	MLR	0.98	0.967	0.086	0.039
			DWLS	1.00	1.013	0.00	0.038
Core research issues	4	176	MLR	0.986	0.957	0.076	0.032
			DWLS	1.00	1.022	0.00	0.032
University support	10	128	MLR	0.818	0.765	0.158	0.082
			DWLS	0.999	0.999	0.13	0.082
Social support	5	202	MLR	0.787	0.573	0.306	0.098
			DWLS	0.961	0.923	0.121	0.093
Mental health	3	205	MLR	1.00	1.00	0.00	0.00
			DWLS	1.00	1.00	0.00	0.00

*Note.* CFI = Comparative Fit Index, TLI = Tucker Lewis Index, RMSEA = Root Mean Square Error of Approximation, SRMR = Standardized Root Mean Square Residual.

The core research issues index involved items related to difficulty in discussing research with colleagues, difficulty in data collection, difficulty in dissemination, and methodological challenges faced while conducting research. The digital literacy index measured the participants ability to access email, virtually access bank accounts, use digital technologies, video conferencing, online file sharing, and learning new technology without the help of a third party.

University support index included the extent of physical, mental, material, and economic support received from university professors and administrators. Furthermore, support received from the university in terms of resources, flexibility in work hours, training, monetary assistance, and financial guidance was also measured. Support received from family, relatives, and peers in terms of physical, mental, material, and economic well-being were included in the social support measure. Mental health index comprised items related to overall mental health, work-life balance, and the amount of happiness one experienced.

**Regression analysis.** Based on significant correlations between variables (see Table 11), multiple regression models were computed using pairwise deletion (lavaan; Rosseel, 2012) to answer each of the above-mentioned research questions (see Table 12). Additionally, regression analysis was also performed on disaggregated datasets based on gender (men and women) and caste (dominant and underprivileged caste). A *post hoc* power analysis using G*Power 3.1 ( Faul *et al*., 2009) was computed for all the models having at least one significant predictor. It was observed that the models had a high power ranging from 0.99-1.00 ( *α*= 0.05) for the differing effect size, sample size, and number of predictors for each model.

**Table 11.** Correlation matrix (participants with a graduate/postgraduate degree).

Variable	*1*	*2*	*3*	*4*	*5*	*6*	*7*	*8*	*9*	*10*
1. Age										
2. People residing in household	-0.02									
3. People residing in household below 18yrs	.22 **	.28 **								
4. People residing in household above 60yrs	0.09	.69 **	.42 **							
5. Caregivers in household	-0.03	.72 **	.32 **	.89 **						
6. Access to independent workspace	-0.03	0.02	-0.02	0.05	0.11					
7. Depend on lab	-0.01	0.01	-0.09	-0.01	0.03	0.07				
8. Human participants	0.02	0.07	0	0.07	0.08	0.01	.36 **			
9. Remote working	0.08	-0.01	0.03	0.05	0.06	.42 **	-0.14	0.1		
10. Stable internet connection	0.06	0.02	-0.02	0	0	.30 **	0	0	.26 **	
11. Disruption in supplies	-0.02	0.06	-0.02	0.05	0.08	-.16 *	.49 **	.23 **	-.22 **	-0.05
12. Core research issues-total	0.04	0.07	0	0.08	0.09	-0.12	.36 **	.28 **	-0.01	-0.06
13. Digital literacy-total	-0.09	0.03	-0.15	-0.03	0.02	.22 **	.23 **	0.06	-0.08	.25 **
14. Difficulty receiving grant	-0.05	0.14	-0.02	0.08	0.09	-0.16	.27 **	0.1	-0.13	-0.13
15. Personal financial stability	-0.01	-0.06	-0.13	-0.1	-0.11	-0.15	0.14	0.14	-0.07	0.04
16. Household financial stability	-0.03	-0.03	-.17 *	-0.07	-0.05	-0.03	.21 *	0.12	-0.1	-0.13
17. Scientific productivity	-0.07	0.01	-.17 *	-0.14	-0.07	-0.04	.18 *	0.13	0.03	-0.02
18. University support-total	-0.06	0.07	0.07	.26 **	.26 **	.26 **	-0.06	0.01	0.13	0.16
19. Social support-total	-0.09	.17 *	-0.09	-0.01	0.09	.24 **	0.06	-0.13	-0.06	.22 *
20. Mental health-total	0	0.07	0.03	0.06	0.02	.26 **	0	-0.07	.17 *	.24 **
21. Stress	0.02	-0.02	-0.05	0.05	0	-0.04	0.06	0.06	-0.11	0.08
Variable	*11*	*12*	*13*	*14*	*15*	*16*	*17*	*18*	*19*	*20*
11. Disruption in supplies										
12. Core research issues-total	.46 **									
13. Digital literacy-total	0.12	-0.04								
14. Difficulty receiving grant	.41 **	.35 **	-0.08							
15. Personal financial stability	.28 **	.37 **	.20 *	.52 **						
16. Household financial stability	.16 *	.41 **	0.14	.39 **	.66 **					
17. Scientific productivity	.21 *	.36 **	.20 *	.31 **	.35 **	.31 **				
18. University support-total	0.02	0.03	-0.03	-0.03	-0.17	-.19 *	-0.01			
19. Social support-total	0.14	0.01	.34 **	0.14	0.16	0.08	.22 **	.35 **		
20. Mental health-total	-0.09	-0.08	-0.1	-0.04	-.21 *	-.31 **	-0.17	.40 **	.21 **	
21. Stress	.17 *	.22 *	.24 **	0.09	.35 **	.26 **	.21 *	0.09	.14 *	-.21 **

*Note*. * *p* < .05. ** *p* < .01.

**Table 12.** Multiple Regression model estimates (participants with a graduate/postgraduate degree).

Research Question	Full Sample		Men		Women		Dominant caste	
	*N*	R ^2^	*N*	R ^2^	*N*	R ^2^	*N*	R ^2^
RQ1- What impacts the ability to continue one’s research during the COVID-19 pandemic?-Core research issues	251	0.230	146	0.280	101	0.117	164	0.207
RQ1- What impacts the ability to continue one’s research during the COVID-19 pandemic?-Logistic issues (Disruption in supply)	261	0.201	148	0.176	109	0.222	172	0.198
RQ3- What impacts researcher’s scientific productivity during the COVID-19 pandemic?	262	0.246	149	0.377	109	0.268	173	0.207
RQ4- What impacts mental health among STEM scientists during the COVID-19 pandemic?- Mental health	261	0.283	149	0.343	108	0.358	172	0.325
RQ4- What impacts mental health among STEM scientists during the COVID-19 pandemic?- Stress	262	0.170	149	0.256	109	0.199	173	0.245

The results showed that a greater difficulty in receiving a grant ( *β* = 0.548, z = 2.082, p = 0.037) and a greater financial insecurity in the household ( *β* = 0.848, z = 2.284, p = 0.022) significantly predicted higher core research issues. Further, greater difficulty in receiving a grant also predicted a higher disruption in lab supplies ( *β* = 0.375, z = 3.569, p = 0.00). It was also observed that an adverse change in scientific productivity was predicted by higher core research issues ( *β* = 0.080, z = 2.835, p = 0.005) and greater support from the university predicted better mental health ( *β* = 0.084, z = 2.628, p = 0.009).

Among men, it was found that household financial instability significantly predicted core research issues ( *β* = 0.987, z = 2.014, p = 0.044) and core research issues predicted an adverse change in scientific productivity ( *β* = 0.115, z = 2.605, p = 0.009). Furthermore, it was noted that a stable internet connection to work remotely ( *β* = 0.677, z = 2.083, p = 0.037) and greater support from the university ( *β* = 0.097, z = 2.093, p = 0.036) predicted better mental health among men.

For women, difficulty in receiving a grant significantly predicted a greater disruption in lab supplies ( *β* = 0.444, z = 2.958, p = 0.003) and, a lower disruption in lab supplies predicted a greater change in one’s scientific productivity ( *β* = -0.282, z = -2.078, p = 0.038). Additionally, greater difficulty in receiving a grant predicted an adverse change in scientific productivity among women ( *β* = 0.374, z = 2.187, p = 0.029).

Greater household financial insecurity predicted more core research issues ( *β* = 0.998, z = 2.309, p = 0.021) among the dominant caste. Further, greater difficulty in receiving a grant also predicted a higher disruption in lab supplies ( *β* = 0.454, z = 2.688, p = 0.007). It was also observed that access to an independent workspace to work from home ( *β* = 0.941, z = 2.625, p = 0.009) and greater support received from the university ( *β* = 0.125, z = 3.126, p = 0.002) significantly predicted better mental health for the dominant caste groups. Due to a small sample size for the underprivileged caste groups (n =55), the correlations were spurious and unreliable to interpret hence, are not reported and included in the analysis. 

For those who had left academia (N = 78) or were thinking about leaving academia (N= 25), due to a small sample size, deducible and reliable results cannot be obtained. Hence, qualitative data will be used as a way to gauge people’s reasons for leaving or considering leaving academia.

### Qualitative results

Sentiment analysis was computed to identify the emotional tone for the qualitative questions included in the survey using RStudio. Furthermore, thematic analysis was conducted to analyse the interview responses using the NVivo 20 software (Released in March 2020).

***Sentiment analysis***

**Figure 3.**
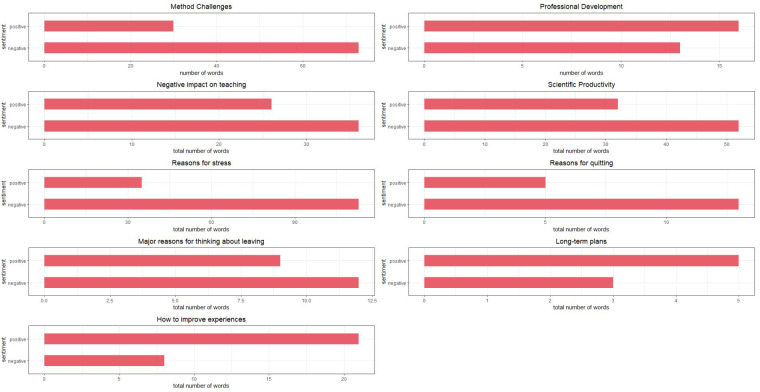
Sentiment analyses results. *Note:* Graphs summarise positive and negative sentiment frequencies of number of words used in a random 6% sample of all descriptive responses provided by survey participants.

Using the ‘bing’ dictionary within the ‘dplyr’ package in R Studio software version 1.4.1717 ( RStudio team, 2021), we explored whether certain qualitative descriptive responses were positively or negatively coded. Specifically, certain emotionally-loaded words were examined and classified at the document level. First, each response for each question was unnested into unigrams (i.e., single words); these words were then assigned positive/negative scores. Next, we further listed the phrases in context using the “keyword in context” function in the “quanteda” package. This function returns words in the immediate context of provided keywords. The main results are summarised in Figure 3.

**Methodological challenges.** We found that the overall sentiment regarding methodological issues were negative, with 73 negatively coded words, and 30 positively coded words. Words such as “broke,” “burden,” and “challenging” were used when participants were asked about methodological challenges.

Words related to “method,” “work,” and “research” were “stopping” their own research work, “remote data collection,” having to change their methods, and not being able to work.

**Professional development**. A total of 16 positive (e.g., accessible, easy, efficient) and 13 negative words (e.g., delays, backward, and burden) were used to describe the changes in professional development. Using the keywords “profession,” “develop,” and “skill,” we found that participants discussed having more time for professional development, and participating in programmes and workshops online. As there were a small number of responses, the impact of the pandemic on professional development is inconclusive.

**Impact on teaching.** To describe the negative effects of the pandemic on teaching, participants used 36 negatively coded terms (e.g., abysmally, anxiety, cheating) and 26 positive ones (e.g., attentive, comfortable, confident). This included discussions about not being comfortable teaching online, lack of lab tutorials and practicals, and lack of feedback and engagement with students. The higher number of negative words indicate the difficulties academic personnel faced while teaching in the pandemic.

**Scientific productivity.** In total, 52 negative words (e.g., delays) and 32 positive words (e.g., engaging, productive) were used to describe changes in scientific productivity. Participants discussed how there were personal and health-based issues, as well as having spent time trying to keep the lab running, rather than on science. In other words, administrative duties and personal issues took away from being productive. On the other hand, once lockdown restrictions were lifted, participants reported being productive. Similarly, one participant discussed re-planning experiments such that a single person could run them. This suggests that researchers’ productivity was affected negatively during the lockdown.

**Mental health.** To describe reasons for stress, 113 negative words (e.g., anxiety, burden, chronic) and 35 positive words were used. Participants described a lack of social interaction, physical activities, being isolated, increase in workload, among other difficulties. The following keywords were used: “stress,” “because,” “anxious,” “anxiety,” “nerv,” “deal,” “mental,” “health.” These yielded responses describing helplessness, death anxiety, and stress related to financial and career trajectories.

**Long-term plans.** Five positive and three negative words were used to describe long-term plans after quitting. These include description of life as uncertain and requiring health and money; further, a few described wanting to switch to industry jobs, and general discontent with academia in India.

**Reasons for leaving.** Overall, 12 negative words and nine positive words were used to describe reasons for leaving academia. Participants described a lack of money, an abundance of bureaucratic and administrative issues and duties. Further, the reduced number of research positions and salary delays were mentioned.

**Recommendations.** Participants used more positive than negative words when asked about recommendations for improving academic experiences. These included transparency, growth opportunities, timely disbursements of funds, improving diversity, and other professional development opportunities.

***Content analysis***

**Reasons for leaving academia.** Participants who had completed their PhD/post doc reported that the major reasons for leaving academia (RQ5; see Table 13) were: reduced funding/money (e.g. “No pay for 6 months due to delays in grant release with no support from the institution to ensure the grant gets released.”), , increased work pressure and workload (e.g. “Too much work, too many research projects + online teaching, constantly on a computer with no time for personal work which started interfering with my health.”), child-care responsibility (e.g. “Need for partial work-from-home options to balance childcare needs.”), and lack of opportunities (e.g. “The pandemic also shut doors to various available research opportunities.”).

**Table 13.** Content analysis to understand a STEM scientist’s decision to leave academia.

Theme	No. of responses	Examples
Money/Funding	5	*“Money”* *“No pay for 6 months due to delays in grant release with no support from the institution to* *ensure the grant gets released.”*
Retired	3	
Lost job	1	*“I lost my job in academia due to inadequate funding from the government funding agency.”*
Lack of job/ research opportunity	2	*“No job opportunity”* *“The pandemic also shut doors to various available research opportunities.”*
Lack of support	1	*“No support from family”*
Bias towards women	1	*“Inherent bias towards women for faculty positions while favouring male candidates without* *transparent hiring process.”*
Personal growth	1	*“Better career prospects and personal growth.”*
Work pressure/ work load	3	*“Too much work, too many research projects + online teaching, constantly on a computer* *with no time for personal work which started interfering with my health.”*
Bad work culture	1	*“I got tired of the very bad work culture at my place.”*
Child care responsibilities	2	*“Need for partial work-from-home options to balance childcare needs.”*
Lack of growth	1	*“no growth.”*
Uncertainty	1	*“Uncertainty of future positions.”*
NA	1	

*Note.* Includes responses from 21 participants; some participants noted multiple reasons.

**Reasons for thinking about leaving academia.** Those ECRs who were thinking about leaving academia (RQ6; see Table 14) mentioned lack of funding (e.g. “Reduced funding”), poor work culture (e.g. “Unfair professional assessment at workplace”), issues related to salary/money (e.g. “Not sure when salary for myself and the other research staff will be released”), lack of support (e.g. “Lack of support from upper management”), higher work pressure and workload (e.g. “A lot of pressure”), bureaucratic issues (e.g. “Unfair, hypocritical, opaque system.”), and lack of job stability/security (e.g. “Lack of job stability”) as reasons.

**Table 14.** Content analysis to understand a STEM scientist’s reason for thinking of leaving academia.

Theme	No. of responses	Examples
Lack of funding	4	*“Reduced funding”*
Poor work culture	3	*“Unfair professional assessment at workplace”*
Salary/Money	5	*“Not sure when salary for myself and the other research staff will be released”*
Bureaucratic issues	4	*“Unfair, hypocritical, opaque system.”*
Lack of support	3	*“Lack of support from upper management”*
Work pressure/ work load	4	*“A lot of pressure”* *“Working on* a contract *is hampering too much. Working 35 hrs per* week is *too much.”*
Job stability/security	3	*“Lack of job stability”*
Recruitment issues	4	*“No recruitment.”*
Growth	2	*“Career development prospects”*
Health	1	
No respect	1	*“No respect for my work.”*
Bad experience	1	*“Due to my poor PhD experience especially during the treatment I received in the* *lockdown time.”*
Resources	1	*“Better medical facility and openness to work independently”*
Awaiting results	1	*“Preparing* for the civil *service examination. Results awaited”*
ok	1	
Nil	1	

*Note.* Includes responses from 22 participants; some participants noted multiple reasons.

***Thematic analysis***

From 341 emails sent to the stakeholders to participate in the interview, the researchers did not receive any response from about 317 participants. Extensive and detailed interviews of 24 stakeholders were conducted to determine their views on the impact of the pandemic on research and the functioning of their organisation and employees. The interviews included a subsample of heads of institutes, representatives from funding agencies, suppliers of scientific equipment and materials, other stakeholders, and ECRs. Due to the unavailability and non-response from the funding agencies and suppliers of scientific equipment, 4 other stakeholders were interviewed (presented as individual case studies).

From the 258 emails sent to the HoIs, 22 funding agencies, and 40 suppliers approached, only 8 HoIs, 3 funders, and 4 suppliers agreed to participate in the interview. Additionally, interviews were conducted with 5 ECRs (from the 21 ECRs who were approached) who elaborated on their reasons for planning to leave academia. Finally, 4 additional stakeholders working in Indian STEM were also interviewed to understand their perspective of the impact on COVID-19 on researchers in India.

For conducting thematic analysis, two researchers coded the interview transcripts. The responses were coded according to the predetermined codes from the interview guide and the literature review conducted for the study while, a few were derived from the data. Following this, through discussion, the researchers came to a consensus on the themes and the codes that the interview responses revealed. The variation in qualitative research designs compounds the intricacies of the saturation question along with the multiple methods of data collection ( Sebele-Mpofu, 2020). Given that predetermined codes (based on the literature review and the questionnaire built on the literature review) were used to analyse the responses in our study, the concept of thematic saturation does not necessarily apply to the current analysis as the study is based on prior research.

***Heads of Institutes (HoIs).*** In understanding the effects of COVID-19 on the institute, HoIs mentioned the impact of the pandemic on research within the institute, digital literacy training for researchers, enforcement of formal policies, challenges associated with virtual mode of communication, deadline-related challenges, and changes in their roles and responsibilities. Furthermore, effects of the pandemic on funding for projects and laboratories, procurement of scientific equipment due to funding, attrition within the institute, influence on hiring, and the impact on scientific productivity were discussed.

The HoI’s also mentioned the change in the proportion of time spent on research supervision, administrative duties, and teaching. In terms of teaching, they discussed the specific requests/challenges experienced by students and teachers along with the shift to online teaching and assessment. Finally, the support received from institutes in terms of flexibility in working hours, connecting to nearby hospitals, and concessions in paying tuition fees for students were highlighted. Additional specialised support for members with childcare responsibilities and specific grievance redressal mechanisms were also provided by the institutes. For a detailed summary of the interview responses with HoIs, refer to the *Extended data*.

**Funding agencies.** Between the three funders interviewed, they fund research in India in the range of USD 14 million, 108 million, and 344 million (only the last figure is for global grants funded). Thus, each operates at a different scale, thematic funding area, and in varying geographical contexts. The major themes that were discussed by the funders included the impact on research output of the institute, effect on future project and funding timelines, changes in funding policies, increase in COVID-19 related research funding, and support to research institutes during the pandemic. For a detailed summary of funding agency responses, refer to the *Extended data*.

**Suppliers of scientific equipment.** The four suppliers who agreed to be interviewed conduct business in products involving high-end imaging platforms, equipment for clinical diagnostics, nuclear research supplies, and telescopes. Each of these operate at a different scale, products, and in varying geographical contexts. The findings for ‘COVID-19 Effects on supply of scientific equipment’ noted the following themes: challenges associated with supply of scientific products, change in demands to COVID-19 testing, diagnostics and research from scientists, and the digital mode of marketing and interactions. Furthermore, the supply was impacted due to change in obtaining funding and payment terms. To understand the challenges faced by suppliers due to the pandemic in detail, refer to the *Extended data*.

**ECRs who had left or were planning to leave academia.** The ECRs were interviewed specifically on their motivations and reasons vis-a-vis leaving or planning to leave academia. The major themes of not being able to do their desired work, difficulty with online teaching, funding difficulties, appraisal and salary issues, overwork, lack of stability and opportunities were highlighted as reasons for leaving or thinking about leaving academia. For a detailed summary of ECR responses, refer to the *Extended data*.

**Other important stakeholders in Indian STEM.** The stakeholders interviewed ranged from research and innovation hubs to companies that bridge the gap between suppliers of scientific equipment and scientists. It also involved platforms that provide communication between life science researchers in India. All the organisations mentioned that research, funding, and supply was directed towards COVID-19 research and efforts during the pandemic. Lockdown restrictions paused research for scientists and supply of materials for research. The pandemic also provided a time for innovation that was geared toward public health and increased virtual communication between researchers and scientists across the country. Suggestions from interviews with these stakeholders focused on increased and equitable funding for research institutes across the country and on timely payments for scientists from funding agencies. Another suggestion was based on reduction of bureaucratic and additional administrative procedures that become an obstacle for scientists in applying for funding. Similarly, scientists and research laboratories face delays due to extensive bureaucratic procedures in taking their research to its final development stage. For a detailed summary of other stakeholder case studies, refer to the *Extended data*.

## Discussion

The purpose of this study was to obtain data to help us understand the comprehensive effect of the COVID-19 pandemic on STEM researchers and stakeholders (suppliers and funders) across India. It was noted from the findings that certain antecedents significantly predicted STEM scientists’ ability to continue research work, teaching, maintain productivity, and mental health during the pandemic. The study highlighted the various challenges faced by early career researchers, and STEM scientists at various positions in their careers during the COVID-19 restrictions in India. 

Extensive research since the onset of the COVID-19 pandemic that focused on its impact on the scientific community, as well as their productivity was conducted. A large number of these studies focused on the disproportionate impact of the pandemic as well as associated lockdown restrictions on female and traditionally underrepresented scientists around the world. These studies have pointed squarely to a larger penalty imposed on female scientists as a result of gendered norms of caregiving and lack of equal opportunities, among others.

Distressingly, our study pointed towards a larger toll on the mental well-being of female early-career researchers (ECRs) in India. Our research focused on ECRs, as they were at a career stage often characterised by job uncertainty, lack of new job opportunities, and a lack of funding ( López-Vergès *et al*., 2021). Thus, that the impact of the pandemic was magnified on this particular sample of researchers. This impact was evident across many fields and found in other large-scale survey work, both during the early stages of the pandemic ( Myers *et al*., 2020), as well as later on ( Morin *et al*., 2021).

First, while there were several studies that found adverse impacts of the pandemic on mental health of scientists ( Chan *et al*., 2020), there were very few that were able to link them to other stressors. For example, Doyle *et al*. (2021) found that physician scientists in the US reported distress on account of increased clinical demands and research delays. Our work suggested that mental health was substantially improved when universities provided support, or scientists had strong social support systems (in the form of relatives, friends, or family), and was also associated with fewer disruptions in research work.

Our finding on the importance of social support, particularly for female ECRs was echoed in work by the National Academy of Sciences ( The impact of COVID-19 on the Careers of Women in Academic Science, Engineering, and Medicine, 2021), which indicated that any social isolation that women face in this regard could damage their well-being and productivity.

Kelly (2021) found a reduction in the time that female scientists were able to devote to research, which mirrored some of the qualitative research findings from our work. However, this meant that they were less ‘visible,’ and therefore less likely to be quoted as experts in the media ( Jones, 2020). Similarly, lack of access to campus facilities was also cited among a large share of scientists in Johnson *et al*. (2021) -- a finding that aligned with the views expressed by heads of institutes / universities as well as other ECRs through interviews.

Gao *et al*. (2021) found that a large number of scientists reported pivoting to COVID-19 research during the pandemic, and our stakeholder interviews confirmed that funders made changes to their strategies to focus on COVID-19. Although quantitative evidence from our study did not suggest that personal or household financial stability played a significant role in mental health concerns or scientific productivity in the sample, research from Australia ( McGaughey *et al*., 2022) and Ireland ( Shankar *et al*., 2021) found that increased career uncertainty and concomitant financial insecurity contributed to greater stress.

Following sections will describe the results in detail based on the proposed research questions.

### Impact on one’s ability to continue research during COVID-19

Specifically, for the individuals who had received a doctorate or a postdoctoral degree, it was observed that those having poor mental health were faced with an increase in core research issues (like methodological challenges, difficulty in data collection and dissemination, staff leaving campus, and difficulty working on campus). Further, greater difficulty in receiving a grant/fellowship led to an increased disruption of procuring lab supplies (slow or compromised supply chains and associated higher costs), and higher digital literacy led to an increase in the number of working hours for professional development (skill development, online courses/webinars, workshops, etc.). Scientists were unable to procure basic lab supplies such as gloves, plastic tips for pipettes, and centrifuge tubes, slowing down or halting research projects ( Woolston, 2021). Among life science trainees based in wet labs it was found that they made use of e-learning software during the lockdown to expand their skills (like, learning a new programming language; Korbel & Stegle, 2020).

In terms of gender, it was observed that for both men and women, poor mental health led to an increase in core research issues. While both genders faced a greater difficulty in receiving a grant or fellowship, it led to a disruption in obtaining lab supplies among men whereas it affected mental health for women.

Taking into account the qualitative responses to the survey questions, it supported the quantitative results suggesting that issues related to money and funding along with health, lack of access to lab, no access to software/hardware, lack of technical support, and absence of research participants were the major methodological challenges faced by the researchers. Further, in terms of professional development individuals mentioned attending conferences and enrolling for courses. 

For individuals who did not have a doctoral degree, the results showed that a greater difficulty in receiving a grant and a greater financial insecurity in the household led to an increase in core research issues. However, participants having a PhD were not affected by difficulties related to financial security. Along with that, a greater difficulty in receiving a grant also gave rise to a higher disruption in procuring lab supplies. A similar trend of difficulty receiving a grant leading to disruption in supplies was observed among participants having a PhD degree. Among men, it was found that household financial instability increased core research issues while for women, difficulty receiving a grant significantly predicted a greater disruption in lab supplies. For individuals belonging to the dominant caste, it was noted that greater household financial insecurity led to more core research issues and greater difficulty in receiving a grant resulted in a higher disruption in lab supplies. It was found that Hispanic and Black undergraduates were more likely than Asians and Whites to delay graduation due to restriction of access to resources and delay in projects (Report 1; Saw *et al*., 2020a). A study noted that PhD students in Brazil belonging to a minority ethnic group were more likely to be financially disadvantaged as compared to white students ( Woolston, 2020b).

### Impact on one’s ability to continue to teach during the COVID-19 pandemic

For those who supervised PhD students, a greater disruption in lab supplies led to a greater impact on their supervisory role. This in turn had an impact on one’s teaching ability. Women (not significant for men) faced a disruption in procuring lab supplies, which affected their supervisory role and faced more difficulty in migrating to online teaching due to lower mental health. This suggested a significant impact of the pandemic on teaching duties of women as compared to men. This is in line with findings from surveys of STEM researchers in Australia. They reported increased challenges in student supervision due to the lack of face-to-face communications, and those with teaching responsibilities had increased teaching workload due to online teaching thus, limiting their research capacity ( EMCR Forum, 2020).

In terms of dominant caste groups, it was observed that being able to manage switching to remote working, better stability in internet connection to work remotely, and lesser disruption in lab supplies had a lower impact on one’s supervisory role. A greater stability in internet connection to work remotely and a better mental health led to a lower difficulty in migrating to online teaching. Due to an unequal sample distribution, any comparison between dominant and underprivileged groups might be difficult to interpret. Additionally, the qualitative results reported a decrease in interaction, money, health, and methodological challenges as the issues having a negative impact on one’s teaching.

### Impact on researcher’s scientific productivity

Susceptibility to greater core research issues (such as difficulty in data collection, dissemination, methodological challenges) led to an adverse change in one’s scientific productivity. An earlier study had shown that many doctoral students and ECRs from the UK were experiencing a negative impact of the lockdown restrictions on their ability to collect data, discuss ideas and findings with colleagues, and disseminate their research findings ( Byrom, 2020). Further, the pandemic had a significant impact on the productivity of early and mid-career researchers in STEM fields in Australia ( EMCR Forum, 2020).

While men’s scientific productivity was affected by external reasons such as, greater research dependency on interactions with human participants and more core research issues (difficulty in data collection, dissemination, methodological challenges), women’s productivity was affected due to personal financial instability and low mental health during the pandemic.

For dominant caste groups, a higher dependency of working in a physical lab for their research, was one of the reasons leading to an adverse change in scientific productivity. Due to an unequal sample distribution, any comparison between dominant and underprivileged groups was difficult to interpret.

Evidence from interviews with ECRs echoed some of these findings. Some of the issues that affected researchers' scientific productivity were uncertainty, loss of time due to COVID-19, decline in scientific output, lack of access to lab, money, mental stress, and change in research field. 

Among the graduate and postgraduate students, adverse changes in scientific productivity were based on higher core research issues (like, difficulty in data collection, dissemination, methodological challenges). Similar trends were also reported among the post-PhD group of participants. While for men greater core research issues led to an adverse change in scientific productivity, for women a greater difficulty in receiving a grant led to an adverse change in productivity. Additionally, lower disruption in lab supplies resulted in a greater change in scientific productivity among women. A study noted that STEM female faculty and students reported facing more problems adapting to remote learning and technological issues as compared to their male colleagues and peers (Report 2; Saw *et al*., 2020b).

### Impact on mental health among STEM scientists

Finally, less difficulty in receiving grants, lower change in scientific productivity, more university and social support led to better mental health among STEM researchers. Specifically, an adverse change in scientific productivity led to lower mental health among researchers which is in line with the findings of an Australian national survey that found the pandemic had a significant impact on mental health and productivity of STEM scientists ( EMCR Forum, 2020). In a study conducted by Ogilvie *et al*. (2020) graduate students mentioned that they received more support from their advisors, professors, and peers in terms of physical and mental well-being ( Ogilvie *et al*., 2020). On the other hand, it was found that researchers having lesser social support networks within and beyond academia tended to struggle with their mental well-being ( Byrom, 2020).

For men, receiving greater university and social support predicted better mental health. For women, difficulty in receiving a grant or fellowship and adverse change in their scientific productivity predicted lower mental health while, receiving higher social support from family, relatives, and peers led to better mental health. These differences bring into light the differential needs and challenges between men and women.

It was also noted that dominant caste groups which received greater social support showed better mental health. Due to an unequal sample distribution, any comparison between dominant and underprivileged groups was difficult to interpret. In terms of the qualitative responses, researchers noted that family and household responsibilities, fear of losing their job, money, health of self and family, and fear of COVID-19 some of the reasons leading to increased stress during the pandemic.

The good mental health of a STEM researcher was a result of greater support received from the university. However, among researchers with a PhD/post-doctoral degree, apart from the importance of university support, difficulty in receiving grants, social support, and change in productivity also affected their mental health. Furthermore, it was noted that a stable internet connection to work remotely and greater support from the university predicted better mental health among men. It was also observed that access to an independent workspace to work from home and greater support received from the university significantly led to better mental health for the dominant caste groups. An ethnographic study had noted that Brahmins and other upper castes dominate in science, medicine, engineering, and academic professions and culturally shape institutions based on their caste identities in India ( Thomas, 2020).

### Reasons for leaving academia and thinking about leaving academia

The section concerning researchers who had left academia and were thinking about leaving academia had a low sample size due to which quantitative inquiry did not lead to any reliable and conclusive results (RQs 5 and 6). Hence, content analysis was conducted on the descriptive responses provided by survey participants for these sections and supplemented by qualitative evidence from interviews with a subsample of ECRs.

Many participants reported issues with money and funding, increased work pressure and workload that were some of the major reasons for leaving academia. Further, a few participants also reported bad work culture, bias towards women, lack of opportunities, loss of job, and child care responsibilities as other reasons for not continuing to work in academia.

Researchers who were thinking about leaving academia mentioned lack of funding, poor work culture, delay in receiving salary, lack of support, high work pressure and workload, job insecurity, and bureaucratic issues as major reasons for the same.

In line with the survey responses, in-depth interviews conducted with ECRs planning to leave or had left academia highlighted similar reasons (RQs 8 and 9). They reported being unable to perform and complete desired work due to the pandemic along with funding difficulties and delays in receiving salary. Further, it was also noted that the issues of teaching online, increased workload, and lack of opportunities and stability were some additional motivators and reasons for leaving and thinking about leaving academia.

### Differential impact of the pandemic among ECRs, Heads of Institutes, suppliers and funders

The survey respondents mentioned ECRs and doctoral students as the ones experiencing the most setbacks in terms of mental, scientific difficulties due to the pandemic. From interviews with HoIs, it was evident that the pandemic impacted scientists in different ways. Lack of access to their research material and laboratories delayed research for some; however, a few scientists were able to return to their labs with precautionary measures. For the HoIs, managing personnel remotely and also on campus once restrictions were lifted were the main challenges of the pandemic. Scenario planning due to the uncertainty of the pandemic was the main challenge and HoIs had to take on new roles to manage this. Managing administrative, supervisory, teaching, research and personnel management tasks were impacted due to the virtual mode of work and the time allotted for each also changed for the HoIs. Ensuring that extensions of grants, additional sources of funding, current funding timelines, and disbursement of salaries was managed during the pandemic was one of the key roles of the HoIs. Mental health of their staff and scientists within the institute and their own mental health was a challenge during the pandemic, even though a few institutes did have counselling support. Virtual coordination of software, hardware, and other research-based support for the scientists was one of the key roles taken up by the HoIs during the pandemic.

For the funding agencies interviewed, they mentioned that current research by the organisations they supported was paused and COVID-19 related research took priority. The organisations supported by the funders were unable to utilise the funds set aside for field work/lab-based work due to lockdown restrictions, but other forms of virtual research still took place. Funders mentioned that committees and boards had to be consulted on the new challenges for funding timelines as presented by the changing nature of the pandemic. The funders interviewed funded organisations, institutes, and individual scientists and the research goals linked to the funding were adapted according to the pandemic. In terms of deadline extensions, funders provided cost and no-cost extensions while also easing the timelines for deliverables required during the funding period. Funding agencies also supported virtual means of research dissemination including workshops, webinars, conferences, and research podcasts with their scientists. This also included virtual meetings with the organisations they supported and regular newsletters on research findings. A suggestion that was highlighted during the interview, was that organisations and institutes across the research spectrum must have a succession plan and a scenario plan in place to ensure minimum disruptions within the organisation's structure due to unforeseeable events in future.

The suppliers of scientific equipment reported a delay in supply of material and equipment owing to lockdown related restrictions on travel within the country and across national borders. Government mandates on manufacturing and supply of material that favour domestic production, especially during the pandemic, impacted suppliers negatively due to added levels of permissions and bureaucratic procedures. Payments for the transportation and delivery of scientific material and equipment were delayed since research institutes were shut due to the lockdown. There were no changes in the type of primary market or target group during the pandemic, and the suppliers moved to virtual means of business through their website and online portals for transactions. However, not everything could be smoothly managed via a virtual medium since equipment needs to be sampled by the scientists or a physical demonstration needs to be completed before an equipment is purchased.

### Policy recommendations that arise from various challenges faced by scientists during the pandemic

We asked participants for their suggestions and base the following policy recommendations on these:

1. Grant management and other administrative duties should be minimised for scientists as it takes away from their research time.

2. Flexible working hours must be adopted by the institute for the researchers to work independently especially during a pandemic when remote working arrangements are the norm.

3. Funding opportunities must be made widely available for the smaller research institutes in the country, and that funding must be disbursed on time from funding agencies.

4. Institutions must have a better environment for growth opportunities, which takes into account researchers’ mental health, work-life balance, and provides holistic support to the researchers, which has gained importance during the pandemic.

5. Institutions must increase job opportunities and prioritise giving learning opportunities to graduates since online education has unfavourably impacted certain courses and skill learning.

6. For women researchers, there should be support in providing day-care, affordable childcare, transport, flexible working hours taking into account the gendered division of labour in the house. Women researchers with children or those who have older people at home have also expressed the need to have flexible working hours as it gets harder to have a work-life balance.

7. The administration should be acquainted with the process of scientific research and there is a need for upskilling in the tech domain to ensure smoother communication and efficient processing of paperwork digitally. An increase in efficiency, especially in the tech domain, of the administration is needed for quick decision-making and to figure out plans in case of changes in the mode of education. 

8. In order to ensure networking and interaction between researchers, there should be more online workshops, conferences, mentorship opportunities and advancement of training to connect with peers.

9. Institutions should extend funding, submission, grant deadlines taking into account lack of access to labs, delay in procuring equipment and reduce the pressure for researchers to keep publishing.

10. Institutes should make efforts to maintain a contingency/reserve fund to deal with similar events in future.

### Implications

Along with providing a detailed understanding on the various challenges faced by researchers in the STEM community, the current study also illuminates the needs of these researchers (such as importance of social and university support) in order to increase their scientific productivity and improve mental health during the pandemic. Noting the impact of the pandemic on mental health of researchers, an important inference from the study is normalising talking about mental health and providing necessary resources to academic personnel to improve their mental health and build coping resources.

The study has many policy implications, such as the need for training and development of STEM scientists in the area of technological skills and digital literacy to provide opportunities for upskilling researchers/professors and being able to transition to hybrid/online working. Furthermore, a necessity to develop standard operating procedures (SOPs) across domains of teaching and research to alleviate losses in the future. Noting the impact of the pandemic on mental health of researchers, an important inference from the study is normalising talking about mental health and providing necessary resources to academic personnel to improve mental hygiene. Finally, setting up reserve funds to provide funding opportunities to researchers in the case of any such future contingency.

Additionally, this research provides the groundwork for addressing the impact of the pandemic on more understudied groups in India such as women and other genders and individuals belonging to the underprivileged caste. Even though many studies have been conducted in countries such as the USA and UK to understand the impact of the pandemic on researchers, especially women and different racial groups, not many studies have highlighted this difference in an Indian context. Finally, this study also gives an idea of how the pandemic affected STEM researchers not only from the perspective of ECRs but also, from a frame of reference of other stakeholders like the funding agencies, suppliers of lab equipment, heads of institutes, and other stakeholders.

Some of the survey participants provided some recommendations to improve researchers' experience in academia and also increase scientific productivity. A reduction in grant management and administrative duties of researchers, availability of funding opportunities, flexibility in working hours, providing additional means of support, and growth opportunities were a few suggestions made by the participants. Additionally, increase in job opportunities and training along with extending submission deadlines and increasing networking among researchers was also reported. Lastly, providing support especially, for women in terms of childcare and transport were highlighted.

### Limitations

Although the current research provides valuable insights into the needs and challenges faced by STEM researchers in India, there are a few limitations of the study. First, the total sample size was small, suggesting that the results cannot be generalised to all the STEM scientists in India.

Secondly, due to the pandemic only digital tools were used to disseminate the survey, making it available to only a select group of individuals having access to a device, internet connection, and possibly belonging to an urban area. Finally, the study lacked equal representation of different caste groups and research disciplines due to which it was difficult to make a comparison between each group regarding the impact of the pandemic. In particular, the study was unable to comment on scientists or ECRs from underprivileged caste groups, who may have faced differing challenges relative to dominant caste group scientists.

### Future directions

Subsequent studies can include a larger sample so that generalizable results are obtained. Additionally, a more representative sample comprising equal participants from different genders, castes, religions, and discipline groups should be made so that comparisons between these can be made. Further, a more inclusive data collection method for the underprivileged groups can be employed in order to have a more representative sample take part in the study.

## Conclusion

In an attempt to evaluate the challenges faced by STEM researchers in India during the pandemic, disruptions in terms of continuing research, impact on scientific productivity, and declining mental health were reported. Quantitative results indicated that ECRs who were susceptible to research issues like difficulties with data collection and dissemination, and methodological challenges had a large impact on their scientific productivity. Furthermore, difficulty in receiving funding led to an increased disruption of procuring lab supplies. It was also noted that better mental health among ECRs was based on less difficulty in receiving grants, lower change in scientific productivity, and more university and social support. Qualitative findings pointed towards issues with funding and increased work pressure as major reasons for leaving academia. Additionally, interviews with diverse stakeholders suggested a disparate effect of the pandemic on institute heads, suppliers, and funders.

## Figures and Tables

**Table 1.** Descriptive statistics of participants with a PhD/post-doctoral degree.

Question	N	Mean	SD	Median	Minimum	Maximum
What is your age (in years)?	291	39.43	7.46	39	26	64
How many children do you have under the age of 6 years?	173	0.67	0.64	1	0	2
In what year did you receive your doctoral degree or complete your postdoctoral training?	275	2013.23	6.89	2015	1985	2021
How many people reside in your household?	269	3.83	1.47	4	0	10
How many people below 18 years of age reside in your household?	270	1	1.01	1	0	5
How many people above 60 years of age reside in your household?	269	0.92	0.96	1	0	5
If no, how many caregivers (apart from you) do you have in your household?	225	0.93	0.82	1	0	4
Since March 2020, when you have been working from home, to what extent did you have access to your own independent workspace? That is, a place where you could work from home with minimal disturbances.	222	7.47	2.8	7	1	11
To what extent does your research depend on working in a physical laboratory?	223	8.49	3.21	10	1	12 *
To what extent does your research involve physical interaction with human participants?	222	7.11	3.45	7	1	12 *
To what extent did you manage to switch to remote working in a virtual environment during the past year?	223	6.55	2.81	6	1	11
To what extent have you had a stable internet connection to work remotely?	222	8.17	2.29	9	1	11
Did you experience any disruption in procuring lab supplies (e.g., slow or compromised supply chains and associated higher costs)?	225	7.83	2.92	9	1	11
To what extent did you experience any difficulty in discussing research work with colleagues?	210	6.1	2.61	6	1	11
To what extent did the frequency of lab meetings change?	209	5.23	2.61	6	1	11
To what extent did you experience difficulty in data collection?	207	7.52	2.94	8	1	11
To what extent did you experience difficulty in dissemination of research findings (e.g., via virtual conferences)?	208	5.99	2.87	6	1	11
To what extent did you have to change from working on your current research topic to COVID-19 related research?	208	4.36	3.31	4	1	11
On an average, was there a change in your number of working hours in terms of research time (e.g., grant writing, data collection, etc.) in the past year?	209	6.47	3.1	6	1	11
To what extent did you face any methodological challenges (e.g., access to laboratory, access to software, access to data, disruption in time-sensitive experiments, etc.) while conducting research during the pandemic?	209	7.59	2.66	8	1	11
To what extent did staff going home affect your research performance?	197	8.03	2.94	8	1	12 *
How many staff did your lab operate with during the lockdown?	197	4.55	3.84	3	1	12 *
To what extent were the staff staying on campus asked to leave?	196	8.68	3.48	10	1	12 *
To what extent were the staff staying on campus able to continue their research work?	195	5.91	4.25	5	1	12 *
To your best knowledge, were the students’ PhD degrees delayed due to the lockdown?	196	8.97	2.81	10	1	12 **
To your best knowledge, were postdoctoral scholars’ training delayed due to the lockdown?	194	9.09	2.82	10	1	12 **
On an average, was there a change in your number of working hours in terms of administration time (e.g., committee meetings, lab administration, etc.) in the past year?	171	6.5	2.98	6	1	11
On an average, was there a change in your number of working hours in terms of professional development (e.g., skill development, online courses/webinars, workshops, etc.) in the past year?	172	6.73	2.85	6	1	11
How many team members (other than yourself) do you have in your lab/research group?	143	9.78	26.09	6	1	300
To what extent are you able to do the following without help from a third party: - Access your email	165	9.85	2.76	11	1	11
To what extent are you able to do the following without help from a third party: - Access your bank account virtually	162	9.67	2.75	11	1	11
To what extent are you able to do the following without help from a third party: - Use digital technologies to work together with colleagues inside and outside your educational organisation.	162	9.59	2.36	11	1	11
To what extent are you able to do the following without help from a third party: - Video conference (e.g., while teaching/during a seminar)	163	9.64	2.34	11	1	11
To what extent are you able to do the following without help from a third party: - Share files (e.g., Dropbox, Google Drive/Classroom)	161	9.86	2.17	11	1	11
To what extent are you able to do the following without help from a third party: - Willing to learn about digital technology for work (e.g., new statistical software)	162	9.08	2.76	11	1	11
Did you face any difficulty in receiving a grant or fellowship?	164	8.18	3.79	9	1	12 *
Since the lockdown in March 2020, to what extent has your personal financial stability been affected?	163	7.57	2.67	6	1	12 **
Since the lockdown in March 2020, to what extent has your household’s financial stability been affected?	164	7.4	2.58	6	1	12 **
How many projects (local/international) have you been a part of as a PI/Co-PI?	158	4.06	24.76	2	0	312
How many projects (local/international) have you been a part of as other collaborator?	155	1.19	2.05	1	0	20
How many new collaborations have you been a part of since March 2020?	151	1.64	3.33	1	0	30
How many local/international conferences have you attended as a delegate?	157	2.1	3.35	1	0	20
How many local/international conferences have you attended as a panellist / speaker?	153	1.85	3.83	1	0	41
How many local/international conferences have you attended as an organizer?	154	0.73	1.63	0	0	15
How many papers have you peer-reviewed?	114	6.54	6.66	4	1	35
How many panels have you served on?	30	3.1	3	3	0	14
Since the lockdown in March 2020, please indicate the number of new articles you published as first/corresponding/lead author (not counting re-submitting the same article).	156	2.6	3.14	2	0	25
Since the lockdown in March 2020, please indicate the number of new articles you published as a co-author).	156	2.13	3.41	1	0	25
Since the lockdown in March 2020, please indicate the number of book chapters you authored/ co-authored.	151	0.81	1.45	0	0	8
Since the lockdown in March 2020, please indicate the number of books you authored/ co-authored.	151	0.23	0.8	0	0	7
How many research grants did you submit or resubmit, since March 2020?	90	2.57	1.95	2	0	12
To what extent do you think your scientific productivity has changed?	161	8.14	2.21	8	1	11
To what extent does your current job/work involve teaching duties?	155	6.81	3.5	7	1	11
Has your current teaching load changed since March 2020?	127	6.98	2.22	6	1	11
How difficult was it for you to migrate to online teaching (when classes were remotely held)?	126	6.4	2.59	6	1	11
To what extent did the pandemic affect your supervisory role?	89	6.75	3.24	8	1	11
Do you think the pandemic had a negative impact on your teaching?	128	5.97	3.36	7	1	11
On an average, was there a change in your number of working hours in terms of teaching time (e.g., preparation, grading) in the past year?	126	7.31	2.3	6	1	11
In terms of your physical and mental health and well-being, how supported have you felt by the following people since March 2020? - Advisor or Major Professor	129	5.83	3.22	6	1	11
In terms of your physical and mental health and well-being, how supported have you felt by the following people since March 2020? - University administrators	147	5.14	3.17	6	1	11
In terms of your material or economic well-being, how supported have you felt by the following people since March 2020? - Advisor or Major Professor	131	5.46	3.29	6	1	11
In terms of your material or economic well-being, how supported have you felt by the following people since March 2020? - University administrators	145	5.32	3.35	6	1	11
To what extent did your institute/university provide access to essential work resources to help continue your research work remotely during the lockdown?	146	6.25	3.52	6	1	11
To what extent did your institute provide flexibility in working hours?	143	7.52	3.32	8	1	11
To what extent did you receive training from your institute to learn new software, which can be operated remotely to continue your teaching or research work?	146	5.12	3.36	6	1	11
To what extent did your university provide loans/ monetary assistance for buying smartphones/ laptop/other hardware equipment (e.g., a microphone)?	144	3.26	3.03	1	1	11
Do you feel that you have received guidance regarding the financial implications of the shutdown to labs and funding?	146	3.1	3.22	1	1	11
To what extent did your university/institute support you at this time?	145	5.79	3.07	6	1	11
In terms of your physical and mental health and well-being, how supported have you felt by the following people since March 2020? - Peers	165	6.95	2.52	6	1	11
In terms of your physical and mental health and well-being, how supported have you felt by the following people since March 2020? - Partner, Family and Relatives	166	8.87	2.38	10	1	11
In terms of your material or economic well-being, how supported have you felt by the following people since March 2020? - Peers	161	5.61	3.35	6	1	11
In terms of your material or economic well-being, how supported have you felt by the following people since March 2020? - Partner, Family and Relatives	163	8.75	2.82	10	1	11
To what extent did your family/relatives support you at this time?	166	8.02	2.89	9	1	11
Please respond to the following with respect to your career in academia: - I feel optimistic about my career in academia	144	7.36	3.24	8	1	11
Please respond to the following with respect to your career in academia: - I feel that my job is highly secure	144	6.44	3.74	7	1	11
How would you rate your overall mental health?	165	7.57	2.44	8	1	11
Did the lockdown have an impact on your physical health? (e.g., sitting at the desk all day, lack of exercise)	165	5.02	2.66	5	1	11
Since the start of 2020, your work-life balance has: Deteriorated - Improved	165	5.04	2.66	5	1	11
To what extent have you felt stressed in the past year?	165	8.15	2.24	8	1	11
Overall, how happy has your life felt to you over the past month?	163	7.39	2.2	8	1	11
How likely are you going to pursue/ continue to pursue a STEM-related academic career? (Thinking of leaving academia)	24	6.92	2.55	7	1	11
How likely are you going to pursue/ continue to pursue a STEM-related academic career? (Left academia)	23	6.65	3.42	7	1	11

*Note.* *12= Not applicable to me, **12= I’m not sure. SD = Standard deviation

**Table 2.** Frequency distribution of participants with a PhD/post-doctoral degree.

Question	Level	N	Frequency
Which gender do you identify as?	Man	297	150
	Woman		141
	Non-Binary/Transgender		0
	Other (self-describe)		0
	Prefer Not to Say		6
What is your marital status?	Single	298	68
	Married		221
	Separated		1
	Divorced		2
	Widowed		2
	Other (self-describe)		1
	Prefer Not to Say		3
Do you have children?	Yes	300	179
	No		121
What is your highest educational level?	Doctorate (MD, PhD)	300	162
	Postdoctoral training		138
From where have you completed your highest level of education?	India (State University)	292	55
	India (Central University)		33
	India (Central Institute)		69
	India (Deemed University)		34
	India (Private University)		8
	University or Institute outside India		93
What is your employment status?	Student	295	16
	Employed (full-time)		244
	Employed (part-time)		10
	Self-employed		5
	Unemployed		14
	Retired		5
	Homemaker		1
Which of the following best describes your current status?	I am currently in academia	295	202
	I am currently in academia, but I am thinking about leaving academia		40
	I have left academia recently (after March 2020)		18
	I had left academia earlier (before March 2020)		7
	I have a PhD but never pursued a career in academia		10
	I had left academia (after March 2020) but I have returned to academia		2
	I had left academia (before March 2020) but I have returned to academia		2
	Other (self-describe)		14
Where are you currently working?	India (State University)	250	21
	India (Central University)		26
	India (Central Institute)		84
	India (Private Institute)		53
	India (R&D Institution)		55
	University or Institute outside India		11
What is your current position?	Teaching/Research Assistant	251	6
	Post Doc or equivalent		47
	Fellowship-sponsored scientist (e.g., Ramalingaswami Re-entry Fellowship)		25
	Adjunct Professor or equivalent		5
	Assistant Professor or equivalent		80
	Associate Professor or equivalent		44
	Full Professor or equivalent		17
	Other (self-describe)		27
In which primary sector are you currently working?	Academic	135	65
	Governmental		24
	For-profit		4
	Not-for-profit		18
	NGO		10
	Industry		9
	Other (self-describe)		5
What is the nature of your position?	Contract-based	271	109
	Permanent		135
	Freelance		8
	Other (self-describe)		19
What religion do you follow?	Hinduism	274	170
	Islam		11
	Christianity		10
	Sikhism		6
	Buddhism		5
	Zoroastrianism		0
	Other (self-describe)		29
	Prefer Not to Say		43
What caste do you belong to, broadly?	General - Brahmin	272	68
	General - Kshatriya		22
	General - Vaishya		12
	General - Other dominant/upper castes		47
	Scheduled Caste (SC)		10
	Scheduled Tribe (ST)		3
	Other Backward Class (OBC)		23
	Other underprivileged/lower caste		0
	None		21
	Prefer Not to Say		57
	Other (self-describe)		9
Are you the primary caregiver in your family?	Yes	269	154
	No		115
What is your primary discipline of research?	Physics	235	10
	Chemistry		4
	Biology		159
	Mathematics		7
	Medicine		21
	Engineering		12
	Information Technology		2
	Humanities and Social Sciences		5
	Other (self-describe)		15
Do you own a personal laptop/desktop to conduct work from home?	Yes	223	187
	University/ Institute provided one		26
	No, had to buy a new one		9
	No		1
Are you currently a part of a lab/research group?	Yes	175	156
	No		19
If applicable, have you had to lay off/furlough any team members? Please select the best option that applies to you	Yes, I have had to temporarily layoff a team member	149	8
	Yes, I have had to permanently layoff a team member		6
	No, and team members are receiving their full salary and not using earned time or vacation time		65
	No, but team members are using up earned time and/or vacation time		12
	No, but team members are being paid less during this time		8
	Other		5
	Not applicable to me		45
Since the lockdown in March 2020, on an average, with respect to the funding for your: - Projects	Funding has increased	164	4
	Funding has decreased		32
	No change in funding		48
	I don’t know		18
	Funding was discontinued		12
	Funding is delayed		50
Since the lockdown in March 2020, on an average, with respect to the funding for your: - Lab	Funding has increased	159	6
	Funding has decreased		45
	No change in funding		35
	I don’t know		25
	Funding was discontinued		14
	Funding is delayed		34
Since the lockdown in March 2020, on an average, with respect to the funding for your: - Department	Funding has increased	159	3
	Funding has decreased		24
	No change in funding		25
	I don’t know		41
	Funding was discontinued		9
	Funding is delayed		27
Since the lockdown in March 2020, on an average, with respect to the funding for your: - Institute	Funding has increased	156	5
	Funding has decreased		46
	No change in funding		23
	I don’t know		51
	Funding was discontinued		9
	Funding is delayed		22
Did you experience any impact of the pandemic on your payroll (on average)?	Delay in receiving the full amount	162	41
	Did not receive the full amount		13
	Received the full amount on time		93
	Received a partial amount		5
	Other (self-describe)		10
Has your fellowship or employment term changed because of COVID-19?	It is uncertain at the moment	164	27
	It has stayed the same		65
	It has been shortened		6
	It has been extended		7
	Not applicable		52
	Other (self-describe)		7
Since the lockdown in March 2020, please indicate if you have served as a peer-reviewer for journal articles?	Yes	164	118
	No		46
Since the lockdown in March 2020, please indicate if you have, served on a review panel for funding?	Yes	164	32
	No		132
Did you submit or resubmit a research grant?	Yes	161	90
	No		71
What are some ways you have been able to maintain productivity within the lab? (Choose all that apply)	A few personnel are still going to lab	75	75
	Focus on data analysis or manuscript writing	115	115
	Collaboration with other labs	52	52
	Diversify the type of research you are working on	51	51
	Grant writing	67	67
	Interacting with collaborators	55	55
	Working on your lab website	18	18
	Other (Self describe)	11	11
Have there been any unexpected silver linings to the COVID crisis? (Check all that apply)	More time to write manuscripts	83	83
	More time to write grants	39	39
	More time with family	93	93
	Other (Self describe)	23	23
What is the level at which you teach?	Junior College/High School	128	0
	Undergraduate		18
	Postgraduate		63
	PhD and higher		47
What is your current teaching load (instructional hours) in hours per week?	Less than 3 hours	128	49
	3 to 6 hours		36
	6 to 12 hours		20
	More than 12 hours		23
Do you supervise PhD students?	Yes	128	89
	No		39
Does your university have online library facilities?	Yes	145	99
	No		46
What has been the primary care format (for dependents) since March 2020?	Splitting time with partner	158	73
	Care by relative		20
	Care by hired help		15
	Independently		43
	Other		7
Did you receive any help for domestic work (e.g., house help, babysitter) in your household?	Yes	164	57
	No		72
	Sometimes		35
Do you experience any chronic health conditions?	Yes	168	45
	No		123
Do you have conditions that leave you immunocompromised?	Yes	167	18
	No		149
Have you ever tested positive for COVID-19?	Yes	168	37
	No		131
Have you received at least one dose of a COVID-19 vaccine?	Yes	167	155
	No		12
Did any members in your household test positive for COVID-19?	Yes	167	57
	No		110
Did you have to step in to help a family member/friend who suffered from COVID-19?	Yes	168	94
	No		74
Did you experience a temporary (or permanent) loss of research personnel who tested positive or displayed COVID-19 symptoms?	Yes	168	90
	No		78
Block 11: People who are thinking of leaving academia			
Since March 2020, have you transferred jobs?	I have transferred from one academic/research institute to another	23	1
	I have transferred from an academic/research institute to industry		1
	I have transferred to a non-academic/research institute		0
	I am thinking about quitting academia		17
	I have quit academia		1
	I am thinking about retiring		1
	Other (self-describe)		2
Have your long-term plans changed due to COVID-19?	Yes	24	20
	No		4
Do you think the pandemic has negatively affected your career prospects?	Yes	24	15
	No		6
	Not sure		3
Do you believe you’ve lost a job offer because of COVID-19?	Yes	24	7
	No		7
	Unsure		10
	Other (self-describe)		0
Block 10: People who have left academia			
Are you planning to return to academia?	Yes	23	6
	No		9
	Maybe		4
	Unsure		4
Since March 2020, have you transferred jobs?	I have transferred from one academic/research institute to another	22	1
	I have transferred from an academic/research institute to industry		2
	I have transferred to a non-academic/research institute		3
	I am thinking about quitting academia		2
	I have quit academia		10
	I am thinking about retiring		0
	Other (self-describe)		4
Have your long-term plans changed due to COVID-19?	Yes	24	18
	No		6
Do you think the pandemic has negatively affected your career prospects?	Yes	23	13
	No		4
	Not sure		6
Do you believe you’ve lost a job offer because of COVID-19?	Yes	23	9
	No		9
	Unsure		4
	Other (self-describe)		1

**Table 7.** Descriptive statistics of participants with a graduate/postgraduate degree.

Question	N	Mean	SD	Median	Minimum	Maximum
What is your age (in years)?	314	29.34	8.26	27	18	92
How many children do you have under the age of 6 years?	84	1.63	2.67	1	0	23
How many people reside in your household?	264	4.63	3.44	4	0	43
How many people below 18 years of age reside in your household?	266	2.12	4.63	1	0	55
How many people above 60 years of age reside in your household?	264	1.66	2.96	1	0	43
If no, how many caregivers (apart from you) do you have in your household?	242	1.91	2.42	2	0	33
Since March 2020, when you have been working from home, to what extent did you have access to your own independent workspace? That is, a place where you could work from home with minimal disturbances.	188	6.29	2.7	6	1	11
To what extent does your research depend on working in a physical laboratory?	188	7.87	3.04	8	1	12 *
To what extent does your research involve physical interaction with human participants?	187	6.76	2.66	6	1	12 *
To what extent did you manage to switch to remote working in a virtual environment during the past year?	187	6.03	2.69	6	1	11
To what extent have you had a stable internet connection to work remotely?	185	6.78	2.67	7	1	11
Did you experience any disruption in procuring lab supplies (e.g., slow or compromised supply chains and associated higher costs)?	189	6.38	2.93	6	1	11
To what extent did you experience any difficulty in discussing research work with colleagues?	179	6.12	2.4	6	1	11
To what extent did the frequency of lab meetings change?	178	6.19	2.67	6	1	11
To what extent did you experience difficulty in data collection?	178	6.85	2.49	6	1	11
To what extent did you experience difficulty in dissemination of research findings (e.g., via virtual conferences)?	178	6	2.5	6	1	11
To what extent did you have to change from working on your current research topic to COVID-19 related research?	178	5.69	2.86	6	1	11
On an average, was there a change in your number of working hours in terms of research time (e.g., grant writing, data collection, etc.) in the past year?	177	6.47	2.53	6	1	11
To what extent did you face any methodological challenges (e.g., access to laboratory, access to software, access to data, disruption in time-sensitive experiments, etc.) while conducting research during the pandemic?	178	6.8	2.44	6	1	11
To what extent did staff going home affect your research performance?	167	7.31	2.63	7	1	12 *
How many staff did your lab operate with during the lockdown?	167	5.84	3.23	6	1	12 *
To what extent were the staff staying on campus asked to leave?	167	7.63	3.12	8	1	12 *
To what extent were the staff staying on campus able to continue their research work?	166	6.48	3.23	6	1	12 *
To your best knowledge, were the students’ PhD degrees delayed due to the lockdown?	167	8.04	2.68	8	1	12 **
To your best knowledge, were postdoctoral scholars’ training delayed due to the lockdown?	166	8.28	2.85	8	1	12 **
On an average, was there a change in your number of working hours in terms of administration time (e.g., committee meetings, lab administration, etc.) in the past year?	161	6.27	2.45	6	1	11
On an average, was there a change in your number of working hours in terms of professional development (e.g., skill development, online courses/ webinars, workshops, etc.) in the past year?	160	6.76	2.45	7	1	11
How many team members (other than yourself) do you have in your lab/ research group?	106	7.37	5.16	6	0	30
To what extent are you able to do the following without help from a third party: - Access your email	159	7.92	3.34	9	1	11
To what extent are you able to do the following without help from a third party: - Access your bank account virtually	154	7.79	3.3	8	1	11
To what extent are you able to do the following without help from a third party: - Use digital technologies to work together with colleagues inside and outside your educational organisation.	156	7.73	3.14	8.5	1	11
To what extent are you able to do the following without help from a third party: - Video conference (e.g., while teaching/during a seminar)	155	7.87	2.98	8	1	11
To what extent are you able to do the following without help from a third party: - Share files (e.g., Dropbox, Google Drive/Classroom)	154	8.06	2.99	9	1	11
To what extent are you able to do the following without help from a third party: - Willing to learn about digital technology for work (e.g., new statistical software)	154	8.05	2.89	9	1	11
Did you face any difficulty in receiving a grant or fellowship?	155	7.67	3.42	8	1	12 *
Since the lockdown in March 2020, to what extent has your personal financial stability been affected?	157	7.59	2.66	7	1	12 **
Since the lockdown in March 2020, to what extent has your household’s financial stability been affected?	156	7.77	2.54	7	1	12 **
How many projects (local/international) have you been a part of as a PI/Co-PI?	132	2.49	4.06	1.5	0	40
How many projects (local/international) have you been a part of as other collaborator?	134	2.25	2.23	2	0	8
How many new collaborations have you been a part of since March 2020?	133	2.17	2.1	2	0	10
How many local/international conferences have you attended as a delegate?	135	2.77	3.22	2	0	20
How many local/international conferences have you attended as a panellist / speaker?	132	2.02	2.18	1	0	7
How many local/international conferences have you attended as an organizer?	131	1.99	2.47	1	0	14
How many papers have you peer-reviewed?	54	4.83	4.92	4	1	25
How many panels have you served on?	46	3.91	2.49	4	0	12
Since the lockdown in March 2020, please indicate the number of new articles you published as first/corresponding/lead author (not counting re- submitting the same article).	130	2.55	3.69	1	0	23
Since the lockdown in March 2020, please indicate the number of new articles you published as a co-author).	130	2.62	5.32	1	0	56
Since the lockdown in March 2020, please indicate the number of book chapters you authored/ co-authored.	128	2.34	5.42	1	0	57
Since the lockdown in March 2020, please indicate the number of books you authored/ co-authored.	128	2.54	6.31	0.5	0	67
To what extent do you think your scientific productivity has changed?	150	7.41	2.27	7	1	11
To what extent does your current job/work involve teaching duties?	146	5.18	3.14	6	1	11
Has your current teaching load changed since March 2020?	98	2.26	0.93	2	1	4
How difficult was it for you to migrate to online teaching (when classes were remotely held)?	98	6.2	2.63	6	1	11
To what extent did the pandemic affect your supervisory role?	29	6.86	2.15	7	3	11
Do you think the pandemic had a negative impact on your teaching?	101	6.57	2.92	7	1	11
On an average, was there a change in your number of working hours in terms of teaching time (e.g., preparation, grading) in the past year?	100	6.48	2.06	6	1	11
In terms of your physical and mental health and well-being, how supported have you felt by the following people since March 2020? - Advisor or Major Professor	135	6.48	2.84	6	1	11
In terms of your physical and mental health and well-being, how supported have you felt by the following people since March 2020? - University administrators	136	6.05	2.97	6	1	11
In terms of your material or economic well-being, how supported have you felt by the following people since March 2020? - Advisor or Major Professor	137	6.13	3.02	6	1	11
In terms of your material or economic well-being, how supported have you felt by the following people since March 2020? - University administrators	135	6.34	3.24	6	1	11
To what extent did your institute/university provide access to essential work resources to help continue your research work remotely during the lockdown?	137	6.76	2.7	7	1	11
To what extent did your institute provide flexibility in working hours?	135	6.63	2.68	6	1	11
To what extent did you receive training from your institute to learn new software, which can be operated remotely to continue your teaching or research work?	136	5.82	2.93	6	1	11
To what extent did your university provide loans/ monetary assistance for buying smartphones/ laptop/other hardware equipment (e.g., a microphone)?	136	4.76	3.12	6	1	11
Do you feel that you have received guidance regarding the financial implications of the shutdown to labs and funding?	133	5.47	3.4	6	1	11
To what extent did your university/institute support you at this time?	135	6.1	2.71	6	1	11
In terms of your physical and mental health and well-being, how supported have you felt by the following people since March 2020? - Peers	207	6.69	2.83	6	1	11
In terms of your physical and mental health and well-being, how supported have you felt by the following people since March 2020? - Partner, Family and Relatives	207	7.81	2.7	8	1	11
In terms of your material or economic well-being, how supported have you felt by the following people since March 2020? - Peers	207	6.14	2.88	6	1	11
In terms of your material or economic well-being, how supported have you felt by the following people since March 2020? - Partner, Family and Relatives	205	7.84	2.68	8	1	11
To what extent did your family/relatives support you at this time?	208	7.4	2.94	7.5	1	11
Please respond to the following with respect to your career in academia: - I feel optimistic about my career in academia	136	6.31	3.47	6	1	11
Please respond to the following with respect to your career in academia: - I feel that my job is highly secure	135	5.79	3.38	6	1	11
How would you rate your overall mental health?	208	6.94	2.54	7	1	11
Did the lockdown have an impact on your physical health? (e.g., sitting at the desk all day, lack of exercise)	208	5.76	2.76	6	1	11
Since the start of 2020, your work-life balance has: Deteriorated - Improved	206	5.93	2.85	6	1	11
To what extent have you felt stressed in the past year?	206	7.21	2.49	7	1	11
Overall, how happy has your life felt to you over the past month?	207	6.78	2.54	7	1	11
How likely are you going to pursue/ continue to pursue a STEM-related academic career? (Thinking of leaving academia)	25	5.84	2.81	6	1	10
How likely are you going to pursue/ continue to pursue a STEM-related academic career? (Left academia)	78	4.82	3.29	5	1	11

*Note.* *12= Not applicable to me, **12= I’m not sure. SD = Standard Deviation

**Table 8.** Frequency distribution of participants with a graduate/postgraduate degree.

Question	Level	N	Frequency
Which gender do you identify as?	Man	315	175
	Woman		134
	Non-Binary/Transgender		2
	Other (self-describe)		0
	Prefer Not to Say		4
What is your marital status?	Single	315	206
	Married		98
	Separated		3
	Divorced		2
	Widowed		2
	Other (self-describe)		0
	Prefer Not to Say		4
Do you have children?	Yes	317	86
	No		231
What is your highest educational level?	Graduation (BA, BSc, BMS, etc.)	313	120
	Post-graduation (MA, MSc, MMS, MBA, MPhil, etc.)		193
From where have you completed your highest level of education?	India (State University)	300	114
	India (Central University)		61
	India (Central Institute)		43
	India (Deemed University)		31
	India (Private University)		41
	University or Institute outside India		10
What is your employment status?	Student	301	117
	Employed (full-time)		97
	Employed (part-time)		14
	Self-employed		23
	Unemployed		31
	Retired		6
	Homemaker		13
Which of the following best describes your current status?	I am currently in academia	303	151
	I am currently in academia, but I am thinking about leaving academia		29
	I have left academia recently (after March 2020)		27
	I had left academia earlier (before March 2020)		46
	I have a PhD but never pursued a career in academia		5
	I had left academia (after March 2020) but I have returned to academia		10
	I had left academia (before March 2020) but I have returned to academia		14
	Other (self-describe)		21
Where are you currently working?	India (State University)	239	58
	India (Central University)		30
	India (Central Institute)		58
	India (Private Institute)		50
	India (R&D Institution)		38
	University or Institute outside India		5
What is your current position?	Teaching/Research Assistant	234	82
	Post Doc or equivalent		16
	Fellowship-sponsored scientist (e.g., Ramalingaswami Re-entry Fellowship)		19
	Adjunct Professor or equivalent		4
	Assistant Professor or equivalent		14
	Associate Professor or equivalent		6
	Full Professor or equivalent		7
	Other (self-describe)		86
In which primary sector are you currently working?	Academic	179	54
	Governmental		34
	For-profit		16
	Not-for-profit		18
	NGO		29
	Industry		10
	Other (self-describe)		18
What is the nature of your position?	Contract-based	259	102
	Permanent		68
	Freelance		49
	Other (self-describe)		40
What religion do you follow?	Hinduism	271	162
	Islam		25
	Christianity		24
	Sikhism		14
	Buddhism		13
	Zoroastrianism		6
	Other (self-describe)		16
	Prefer Not to Say		11
What caste do you belong to, broadly?	General - Brahmin	267	69
	General - Kshatriya		34
	General - Vaishya		38
	General - Other dominant/upper castes		36
	Scheduled Caste (SC)		18
	Scheduled Tribe (ST)		4
	Other Backward Class (OBC)		24
	Other underprivileged/lower caste		9
	None		13
	Prefer Not to Say		21
	Other (self-describe)		1
Are you the primary caregiver in your family?	Yes	269	128
	No		141
What is your primary discipline of research?	Physics	196	26
	Chemistry		16
	Biology		88
	Mathematics		8
	Medicine		11
	Engineering		17
	Information Technology		8
	Humanities and Social Sciences		12
	Other (self-describe)		10
Do you own a personal laptop/desktop to conduct work from home?	Yes	188	119
	University/ Institute provided one		21
	No, had to buy a new one		31
	No		17
Are you currently a part of a lab/research group?	Yes	165	113
	No		52
If applicable, have you had to lay off/furlough any team members? Please select the best option that applies to you	Yes, I have had to temporarily layoff a team member	109	23
	Yes, I have had to permanently layoff a team member		10
	No, and team members are receiving their full salary and not using earned time or vacation time		14
	No, but team members are using up earned time and/or vacation time		8
	No, but team members are being paid less during this time		10
	Other		4
	Not applicable to me		40
Since the lockdown in March 2020, on an average, with respect to the funding for your: - Projects	Funding has increased	153	18
	Funding has decreased		26
	No change in funding		24
	I don’t know		43
	Funding was discontinued		17
	Funding is delayed		25
Since the lockdown in March 2020, on an average, with respect to the funding for your: - Lab	Funding has increased	142	11
	Funding has decreased		25
	No change in funding		26
	I don’t know		39
	Funding was discontinued		18
	Funding is delayed		23
Since the lockdown in March 2020, on an average, with respect to the funding for your: - Department	Funding has increased	142	7
	Funding has decreased		29
	No change in funding		27
	I don’t know		40
	Funding was discontinued		22
	Funding is delayed		17
Since the lockdown in March 2020, on an average, with respect to the funding for your: - Institute	Funding has increased	141	8
	Funding has decreased		30
	No change in funding		27
	I don’t know		38
	Funding was discontinued		22
	Funding is delayed		15
Did you experience any impact of the pandemic on your payroll (on average)?	Delay in receiving the full amount	153	56
	Did not receive the full amount		24
	Received the full amount on time		49
	Received a partial amount		12
	Other (self-describe)		12
Has your fellowship or employment term changed because of COVID-19?	It is uncertain at the moment	158	33
	It has stayed the same		47
	It has been shortened		22
	It has been extended		18
	Not applicable		27
	Other (self-describe)		11
Since the lockdown in March 2020, please indicate if you have served as a peer-reviewer for journal articles?	Yes	155	57
	No		98
Since the lockdown in March 2020, please indicate if you have served on a review panel for funding?	Yes	153	47
	No		106
Did you submit or resubmit a research grant?	Yes	151	53
	No		98
What are some ways you have been able to maintain productivity within the lab? (Choose all that apply)	A few personnel are still going to lab	61	61
	Focus on data analysis or manuscript writing	73	73
	Collaboration with other labs	31	31
	Diversify the type of research you are working on	41	41
	Grant writing	23	23
	Interacting with collaborators	29	29
	Working on your lab website	19	19
	Other (Self describe)	13	13
Have there been any unexpected silver linings to the COVID crisis? (Check all that apply)	More time to write manuscripts	62	62
	More time to write grants	37	37
	More time with family	74	74
	Other (Self describe)	25	25
What is the level at which you teach?	Junior College/High School	101	27
	Undergraduate		37
	Postgraduate		30
	PhD and higher		7
What is your current teaching load (instructional hours) in hours per week?	Less than 3 hours	98	25
	3 to 6 hours		31
	6 to 12 hours		34
	More than 12 hours		8
Do you supervise PhD students?	Yes	101	29
	No		72
Does your university have online library facilities?	Yes	129	70
	No		59
What has been the primary care format (for dependents) since March 2020?	Splitting time with partner	204	54
	Care by relative		56
	Care by hired help		25
	Independently		53
	Other		16
Did you receive any help for domestic work (e.g., house help, babysitter) in your household?	Yes	204	79
	No		100
	Sometimes		25
Do you experience any chronic health conditions?	Yes	209	70
	No		139
Do you have conditions that leave you immunocompromised?	Yes	207	52
	No		155
Have you ever tested positive for COVID-19?	Yes	208	83
	No		125
Have you received at least one dose of a COVID-19 vaccine?	Yes	207	157
	No		50
Did any members in your household test positive for COVID-19?	Yes	205	97
	No		108
Did you have to step in to help a family member/friend who suffered from COVID-19?	Yes	207	120
	No		87
Did you experience a temporary (or permanent) loss of research personnel who tested positive or displayed COVID-19 symptoms?	Yes	208	96
	No		112
Block 11: People who are thinking of leaving academia			
Since March 2020, have you transferred jobs?	I have transferred from one academic/research institute to another	25	6
	I have transferred from an academic/research institute to industry		2
	I have transferred to a non-academic/research institute		5
	I am thinking about quitting academia		9
	I have quit academia		2
	I am thinking about retiring		0
	Other (self-describe)		1
Have your long-term plans changed due to COVID-19?	Yes	25	15
	No		10
Do you think the pandemic has negatively affected your career prospects?	Yes	25	18
	No		4
	Not sure		3
Do you believe you’ve lost a job offer because of COVID-19?	Yes	25	13
	No		8
	Unsure		4
	Other (self-describe)		0
Block 10: People who have left academia			
Are you planning to return to academia?	Yes	80	13
	No		39
	Maybe		17
	Unsure		11
Since March 2020, have you transferred jobs?	I have transferred from one academic/research institute to another	81	7
	I have transferred from an academic/research institute to industry		10
	I have transferred to a non-academic/research institute		19
	I am thinking about quitting academia		8
	I have quit academia		25
	I am thinking about retiring		7
	Other (self-describe)		5
Have your long-term plans changed due to COVID-19?	Yes	82	46
	No		36
Do you think the pandemic has negatively affected your career prospects?	Yes	81	40
	No		22
	Not sure		19
Do you believe you’ve lost a job offer because of COVID-19?	Yes	80	30
	No		25
	Unsure		23
	Other (self-describe)		2

## Data Availability

Open Science Framework: Assessing the Impact of COVID-19 on STEM (Science, Technology, Engineering, Mathematics) Researchers in India. https://doi.org/10.17605/OSF.IO/MVXDB ( Mehta *et al*., 2022). This project contains the following underlying data: IA_abovephd-analysis.csv IA_belowphd-analysis.csv Interview transcripts.zip Open Science Framework: Assessing the Impact of COVID-19 on STEM (Science, Technology, Engineering, Mathematics) Researchers in India. https://doi.org/10.17605/OSF.IO/MVXDB ( Mehta *et al*., 2022). This project contains the following extended data: India_Alliance_-_Survey_-_English.docx India_Alliance_Questionnaires.docx (the semi-structured interview schedule) Qualitative analysis_interviews.docx (analysed qualitative responses from the participants) Data are available under the terms of the Creative Commons Zero "No rights reserved" data waiver (CC0 1.0 Public domain dedication).
