# Supplementary material for: Assessing the impact of COVID-19 on STEM (science, technology, engineering, mathematics) researchers in India
Source: Wellcome Open Res. 2023 Jul 11;7:157. Originally published 2022 May 20. [Version 2] doi: 10.12688/wellcomeopenres.17853.2 (PMC10457572; doi:10.12688/wellcomeopenres.17853.2)
Supplement: Peer review history

# Peer review: Reviewer response for version 2

**DOI:** 10.21956/wellcomeopenres.21674.r62572

**Published:** 2023-08-25

**Authors:** Katherine Christian

**Affiliations:** 1 Queensland University of Technology, Brisbane, Australia

In my initial review, I commented that "I have no issues about the research funding". I apologise as this should have read research "findings" and these findings appear to be reasonable in light of the data collected.

 So saying, and relevant to this comment, my main concern remain true. The authors' main areas of concern within the findings are **likely to be *always* present in the STEM environment; they have been exacerbated by the COVID pandemic, not caused by the pandemic. Stress, lack of funding, intention to leave and lack of work life balance are endemic for researchers in STEM worldwide, and particularly for early career researchers. This means that most of the recommendations offered by the authors as a solution to the problems are relevant for people working in STEM at all times, not just for those STEM fields in a pandemic, as presented.

Is the work clearly and accurately presented and does it cite the current literature?

Yes

If applicable, is the statistical analysis and its interpretation appropriate?

I cannot comment. A qualified statistician is required.

Are all the source data underlying the results available to ensure full reproducibility?

Yes

Is the study design appropriate and is the work technically sound?

Yes

Are the conclusions drawn adequately supported by the results?

Partly

Are sufficient details of methods and analysis provided to allow replication by others?

Yes

Reviewer Expertise:

Difficulties of early career researchers in STEMM in Australia

I confirm that I have read this submission and believe that I have an appropriate level of expertise to confirm that it is of an acceptable scientific standard.

# Peer review: Reviewer response for version 2

**DOI:** 10.21956/wellcomeopenres.21674.r62571

**Published:** 2023-08-11

**Authors:** Vineeta Bal

**Affiliations:** 1 IISER Pune: Indian Institute of Science Education Research Pune, Pune, Maharashtra, India

No further changes needed.

Is the work clearly and accurately presented and does it cite the current literature?

Yes

If applicable, is the statistical analysis and its interpretation appropriate?

I cannot comment. A qualified statistician is required.

Are all the source data underlying the results available to ensure full reproducibility?

Yes

Is the study design appropriate and is the work technically sound?

Yes

Are the conclusions drawn adequately supported by the results?

Partly

Are sufficient details of methods and analysis provided to allow replication by others?

Yes

Reviewer Expertise:

NA

I confirm that I have read this submission and believe that I have an appropriate level of expertise to confirm that it is of an acceptable scientific standard.

# Peer review: Reviewer response for version 1

**DOI:** 10.21956/wellcomeopenres.19769.r56170

**Published:** 2023-05-03

**Authors:** Vineeta Bal

**Affiliations:** 1 IISER Pune: Indian Institute of Science Education Research Pune, Pune, Maharashtra, India

The article by Mehta et al. aims to evaluate impact of Covid-19 on not only STEM researchers, but also additional stake holders in India. Inclusion of stakeholders other than researchers adds value to this work. While researchers from early career stage to older ones, heads of institutions are the expected inclusions; funders, equipment suppliers are also included to provide a more rounded picture of the impact. In addition to equipment supply, researchers were also hampered by extensive delays in the supply of laboratory chemicals and reagents. It is not clear whether they were included or not.

 In the **introduction** , the authors have provided an extensive background for their research. There have been many manuscripts/papers published reviewing impact of Covid-19 on researchers at all stages of their career from early pandemic days onwards. Work from India is very limited and that in itself is good enough justification for a systematic effort to understand the situation, highlight difficulties and suggest possible solutions. Solutions of long-term nature to avoid repetition of the crisis are the most valuable ones for any country. However, such a clear set of recommendations for the future are not coming forth.

 The **methodology** consisted of a long questionnaire primarily for early career researchers and in depth personal interviews of other stakeholders. While there was no specific effort made to include women researchers in India in this study, fortunately enough women participants are included to gauge specific impact, if any, of Covid-19 on their work and career. In contrast, other underprivileged categories of researchers such as scheduled castes, scheduled tribes, religious minorities, LGBTIQ+ individuals have not been well represented. Two points of criticism here. 'Suppressed class' may not be the best phrase to use for individuals from under privileged categories. Two, while 'social distancing' was a common term used during Covid-19 pandemic worldwide, in specific Indian context this phrase has a distinct contextual meaning - a behaviour which involves treating people as outcastes or 'untouchables'; and hence 'physical distancing' would have been a better way to express the practice followed during this period.

 Text on methodological details is repetitive and some editing would have made it more concise and better to read.

 There is a mention of compensation provided to each participant. It is assumed it was Rs. 100/- per person. But at one place it is mentioned as Rs. 1000/-.

 Questionnaire was meant for early career researchers, however, at least one researcher aged 64 years participated in the survey (Appendix, Table 1, data on age). This suggests there was possibly no clear, strict definition of 'early career'.

 There is no mention whether the questionnaire was pre-tested. While seasoned researchers do it as a matter of course, a statement to the effect was desirable.

 So called 'suppressed class' individuals are underrepresented. However, in non-PhD category of respondents their number is 56. It would have been worthwhile to provide qualitative information about their specific problems if they were there. The authors have given a lot of emphasis on doing quantitative analysis of the data collected from the questionnaire, by developing specific algorithms but this survey encompasses socio-culturally different participants as well and hence it feels that an opportunity to evaluate comparative qualitative experiences of underprivileged participants is missed in the effort.

 More specifically, in addition to the earlier comment on the underprivileged respondents, the **results** section includes minimum details of the personal interviews in the main text. Only passing statements are mentioned. Instead of checking the details in the supplementary information (extended information) it would have been better to get a sense of findings in the main text. Importance of personal interviews to the whole picture is diminished because of this near-absence of information in the main text.

 Analysis of data from questionnaires is presented as many figures and tables. While providing details is important some details occupy a lot more space than they deserve. For example Figure 3 describing 'sentiment analysis'. Firstly it is unclear what it conveys in each panel. Secondly, the axes are unreadable while the bars occupy huge space in each panel. Thirdly, how meaningful is this level of quantification?

 Based on quantitative data statistical analysis is done, however, clear trends, impressions and take home messages from the data are possibly getting lost. They are not clearly described in the text. Hence those who cannot clearly read the tables and interpret the data as presented are unlikely to get anything much out of the extensive work done by the authors.

 Statistical analysis, for example, has thrown up some differences between men and women researchers. Is there a social significance of these findings? Is that going to help in policy related decisions? While authors are not expected to be the advisors on public health policy, if there were clear meaningfully different impact of Covid-19 on men versus women it should have been made apparent. Even after reading the whole text one is left searching for clear observations.

 In **discussion** , policy recommendations arising out of the comments of the respondents are mentioned. Apart from a significant impact on the researchers' mental health during Covid-19 due to extreme uncertainty and stress, no major new point emerges.

 In conclusion, the work done is sound in terms of planning and execution including data collection from early career researchers. Data analysis is mired with the use of complex statistical methods making it a manuscript for statisticians than other researchers. Personal interviews and comments from heads of the institutions and others would have been very useful to include in the main text, and shift many tables and re-analysis of primary data as extended data. Regardless of these limitations it is clear that early career researchers, especially those without a permanent job in hand, are affected much more than those with a permanent job in hand. Men and women both are affected badly, possibly somewhat on different fronts; but mental health issues are very significant and may have lasting impact on the researchers. Funding delays, delays in equipment procurement (and reagents and chemicals procurement) were critical in paralysing working conditions. Having data on Indian situation during Covid-19 pandemic should provide the basis for prevention of future disruptions of this kind.

 (My lack of expertise in understanding statistical methodology has been mentioned above.)

Is the work clearly and accurately presented and does it cite the current literature?

Yes

If applicable, is the statistical analysis and its interpretation appropriate?

I cannot comment. A qualified statistician is required.

Are all the source data underlying the results available to ensure full reproducibility?

Yes

Is the study design appropriate and is the work technically sound?

Yes

Are the conclusions drawn adequately supported by the results?

Partly

Are sufficient details of methods and analysis provided to allow replication by others?

Yes

Reviewer Expertise:

NA

I confirm that I have read this submission and believe that I have an appropriate level of expertise to confirm that it is of an acceptable scientific standard, however I have significant reservations, as outlined above.

# Peer review: Reviewer response for version 1

**DOI:** 10.21956/wellcomeopenres.19769.r56171

**Published:** 2023-04-20

**Authors:** Katherine Christian

**Affiliations:** 1 Queensland University of Technology, Brisbane, Australia

This paper has been prepared thoughtfully and is well written. The literature review is thorough and up to date and has brought several papers of which I was unaware to my attention. Given the funding body and the platform for publication I am surprised at the omission of the Wellcome Trust report "What researchers think about the culture they work in".

 The research appears to have been carried out in a suitable manner. I note the questionnaire is very long which will not have helped the response rate. I would like to see the base questions for the interview - or to know they exist.

 While I have no issues about the research funding, my main concern is that the authors do not point out, or perhaps even realise as it is not mentioned at all, that their areas of concern are *always* present in the STEM environment; they have been exacerbated by the COVID pandemic, not caused by the pandemic. Stress, lack of funding, intention to leave and lack of work life balance are endemic for researchers in STEM, and particularly for early career researchers.

 Following this, it is not realistic for most academic researchers to expect they will continue in a research career (although they do expect to). There are not enough jobs - or enough funding. The fact they were forced to seek alternate career opportunities during COVID was probably a benefit for them.

 It would be interesting to see the views of PhD students and early career researchers compared with the more senior researchers. It would also be interesting to know whether there is empathy for the difficulties of the researchers from the HoIs or funding bodies.

 While I am not conflicted in any way, I note that I have published on the difficulties for researchers in STEMM disciplines in Australia.

 I further note I do not have the expertise to comment on the statistical analysis although I have no reason to doubt its accuracy.

Is the work clearly and accurately presented and does it cite the current literature?

Yes

If applicable, is the statistical analysis and its interpretation appropriate?

I cannot comment. A qualified statistician is required.

Are all the source data underlying the results available to ensure full reproducibility?

Yes

Is the study design appropriate and is the work technically sound?

Yes

Are the conclusions drawn adequately supported by the results?

Partly

Are sufficient details of methods and analysis provided to allow replication by others?

Yes

Reviewer Expertise:

Difficulties of early career researchers in STEMM in Australia

I confirm that I have read this submission and believe that I have an appropriate level of expertise to confirm that it is of an acceptable scientific standard, however I have significant reservations, as outlined above.
